# Ferroptosis and Cuproptosis in Cancer and Neurodegeneration: A Comprehensive Review of Modulation by Iron and Copper Chelators and Related Agents

**DOI:** 10.3390/biom16030348

**Published:** 2026-02-26

**Authors:** Iogann Tolbatov, Alessandro Marrone

**Affiliations:** 1 Department of Chemical, Physical, Mathematical and Natural Sciences, University of Sassari, 07100 Sassari, Italy; 2 Department of Pharmacy, University “G. D’Annunzio” of Chieti-Pescara, Via dei Vestini 31, 66100 Chieti, Italy; amarrone@unich.it

**Keywords:** ferroptosis, cuproptosis, cancer, neuroprotection, metal chelators

## Abstract

Dysregulation of iron and copper homeostasis is a pivotal driver of regulated cell death through two distinct yet interconnected modalities: ferroptosis and cuproptosis. This comprehensive review evaluates the therapeutic modulation of these metal-driven pathways within a dual paradigm: their deployment as a cytotoxic weapon in oncology and their inhibition for neuroprotection. We synthesize evidence ranging from small-molecule synergy to advanced nanomedicine, examining how the interplay between iron and copper governs cellular fate in resistant malignancies and neurodegenerative diseases such as Parkinson’s disease and Multiple Sclerosis. In oncology, bimetallic nanoplatforms and CRISPR-Cas9 nano-ionophores exploit “iron addiction” and metabolic vulnerabilities to induce fatal lipid peroxidation and FDX1-mediated proteotoxic stress, often by circumventing efflux transporters like ATP7A/B. Conversely, neuroprotective strategies focus on site-specific chelation, utilizing brain-penetrant molecules like SK4 (targeting the LAT1 transporter) and radical trapping antioxidants like Cu^II^(atsm). Importantly, we elucidate the “iron trap” mechanism, where copper deficiency inactivates multicopper ferroxidases—including ceruloplasmin and hephaestin—thereby triggering iron-dependent ferroptosis. Our analysis reveals a self-amplifying cycle of oxidative damage driven by metal-induced ATP depletion and glutathione exhaustion. By delineating the molecular machinery of iron and copper metabolism, this article provides a roadmap for leveraging regulated cell death to overcome apoptosis resistance in cancer and preserve neural integrity in chronic degeneration.

## 1. Introduction

The intricate relationship between metal ion homeostasis and regulated cell death (RCD) has become a critical area of investigation across various human pathologies, including cancer and neurodegeneration [1,2]. Iron (Fe) and copper (Cu), as essential biometals, are vital for biological processes, yet their dysregulation can initiate distinct forms of RCD known as ferroptosis and cuproptosis [3]. These metal-driven modalities of cell death present novel therapeutic targets [4,5], particularly in contexts where conventional treatments fail due to apoptosis resistance (Table 1).

Ferroptosis, initially characterized in 2012 [6], is an iron-dependent form of non-apoptotic cell death driven by the unrestricted accumulation of lipid peroxides (LOOH) and subsequent membrane damage [7,8]. The biochemical identity of ferroptosis is distinct from other reactive oxygen species (ROS)-associated death pathways, which involve generic damage to DNA or proteins. Ferroptosis is uniquely defined by the iron-dependent propagation of lipid peroxyl radicals within a specific membrane context. It is critical to distinguish ferroptosis from generic oxidative stress; while species such as H_2_O_2_ and O_2_•^−^ may act as upstream initiators, the execution of ferroptosis is uniquely driven by the iron-dependent propagation of lipid peroxyl radicals within the phospholipid bilayer [7]. Consequently, “ferroptosis-associated oxidative stress” refers specifically to this membrane-localized radical chain reaction rather than non-specific cytosolic damage. This process is fundamentally triggered by an increase in the labile iron pool (LIP), specifically bioactive Fe^2+^. This iron catalyzes the production of highly toxic lipid peroxyl radicals via the Fenton reaction, rather than generic ROS like H_2_O_2_ [6,8].

Subsequently, these radicals drive the peroxidation of polyunsaturated fatty acids (PUFAs), resulting in catastrophic membrane damage [8]. This stage is enzymatically primed by acyl-CoA synthetase long-chain family member 4 (ACSL4) and lysophosphatidylcholine acyltransferase 3 (LPCAT3), which define the specificity of the ferroptotic membrane lipid context [9,10]. Importantly, the biochemical origin of the lethal ferroptotic signal is not found in generic lipid damage but is specifically rooted in the peroxidation of polyunsaturated fatty acid-containing phosphatidylethanolamines (PUFA-PEs). ACSL4 exhibits a high substrate preference for long-chain PUFAs, such as arachidonic and adrenic acids, which are subsequently esterified into phospholipids by LPCAT3 [11]. These PUFA-PEs serve as the primary substrates for both iron-catalyzed non-enzymatic Fenton chemistry and enzymatic peroxidation by lipoxygenases. A critical distinction exists between these two modes of initiation. Enzymatic peroxidation is primarily mediated by the arachidonate lipoxygenase (ALOX) family, specifically 12/15-ALOX, which utilizes iron as a cofactor to catalyze the stereospecific oxygenation of PUFA-PEs [12]. This enzymatic phase is often considered the initiator of the ferroptotic signal. Conversely, non-enzymatic lipid peroxidation occurs when the LIP reacts with existing LOOH via Fenton-like chemistry. This produces highly reactive alkoxyl and peroxyl radicals that drive a stochastic, self-amplifying chain reaction. Thus, while ALOX enzymes provide the initial biochemical specificity, the eventual catastrophic membrane failure results from a dual-layered process where enzymatic initiation is compounded by non-enzymatic, iron-driven propagation.

The process is enzymatically regulated by the phospholipid hydroperoxide glutathione peroxidase 4 (GPX4), which utilizes reduced glutathione (GSH) to reduce LOOH into non-toxic lipid alcohols (LOH). While the GSH/GPX4 axis is central, it does not act in isolation; the execution of ferroptosis is also governed by parallel GPX4-independent defense systems, including the FSP1-CoQ10 axis and mitochondrial DHODH [13,14]. The FSP1-CoQ10 axis operates primarily at the plasma membrane, where ferroptosis suppressor protein 1 (FSP1) functions as an oxidoreductase that utilizes NADPH to reduce coenzyme Q_10_ (ubiquinone) to ubiquinol (CoQ_10_H_2_), a potent radical-trapping antioxidant that directly neutralizes lipid peroxyl radicals. Similarly, dihydroorotate dehydrogenase (DHODH) provides a localized defense within the mitochondrial inner membrane by facilitating the reduction of ubiquinone to ubiquinol during the process of pyrimidine synthesis. Consequently, the suppression of GPX4 activity or the depletion of the GSH pool are key events leading to ferroptosis [7], but the ultimate transition to cell death is often contingent upon the simultaneous exhaustion of these non-canonical, CoQ10-mediated repair pathways [13,14].This specificity underscores that ferroptosis is a regulated, substrate-specific event where the iron-copper nexus specifically targets primed membrane lipids to drive rupture.

Cuproptosis, a more recently defined modality [15], is triggered by the accumulation of excess intracellular copper ions. The transition from physiological copper signaling (cuproplasia) to cuproptosis represents a failure of cytosolic buffering systems, including metallothioneins and glutathione, to sequester excess copper [15]. This accumulation leads to proteotoxic stress, characterized by the targeted aggregation of lipoylated proteins (specifically dihydrolipoamide S-acetyltransferase, DLAT) involved in the mitochondrial Krebs cycle, culminating in cell death [16]. The upstream regulator ferredoxin 1 (FDX1) facilitates the reduction of Cu^2+^ to the highly toxic monovalent ion Cu^+^. This generation of Cu^+^ is critical for inducing protein aggregation. Furthermore, it triggers the subsequent loss of Fe-S cluster proteins, culminating in metabolic collapse [17].

The biochemical boundary between cuproplasia and cuproptosis is thus defined by a metabolic inflection point: while cuproplasia represents a physiological extension of copper-dependent enzymatic activity that supports proliferation, the cuproptotic shift occurs when the rate of FDX1-mediated Cu^2+^ reduction surpasses the mitochondrial buffering capacity [5,15]. This results in the qualitative transition of DLAT from its functional, lipoylated monomeric form to high-molecular-weight toxic oligomers, accompanied by the irreversible degradation of Fe-S cluster integrity in respiratory complexes.

While the mitochondrial matrix is the primary execution site for cuproptosis, the initiation of this pathway is contingent upon a failure of cytosolic copper sequestration. Under homeostatic conditions, copper chaperones and buffering systems, including metallothioneins and GSH, maintain the labile copper pool at negligible levels. Cuproptosis represents a compartmental breakdown where excessive mitochondrial import or the exhaustion of cytosolic buffers allows copper to reach the FDX1-regulated machinery [17,18]. The precise role of FDX1 in this process remains a subject of active investigation; it is currently debated whether FDX1 acts primarily as a catalyst for Cu^2+^ to Cu^+^ reduction, as a structural scaffold for protein–protein interactions, or indirectly through its control over the lipoylation machinery [15]. Furthermore, while FDX1 is the primary known regulator, the existence of FDX1-independent cuproptotic pathways in specific tumor types remains an open question. Acknowledging these unresolved biochemical distinctions is essential for a rigorous understanding of the cuproptosis execution phase.

**Figure 1:**
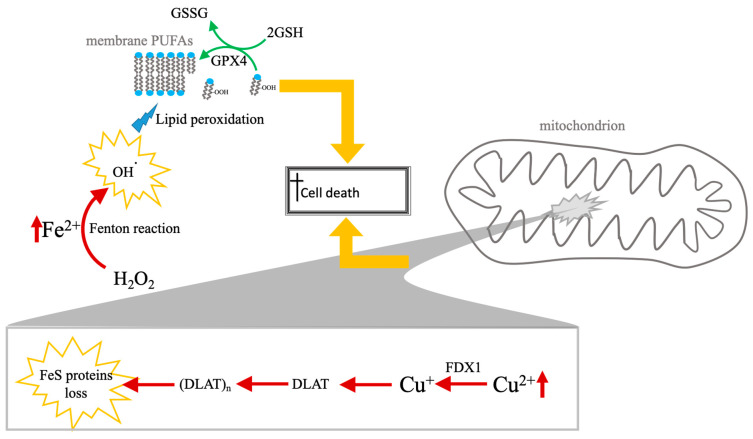
Comparative mechanisms of ferroptosis and cuproptosis. The diagram illustrates the distinct biochemical pathways leading to RCD cell death via iron and copper accumulation. Left panel (ferroptosis): cell death is driven by the accumulation of labile ferrous iron (Fe^2+^), which catalyzes the production of hydroxyl radicals (•OH) via the Fenton reaction. These radicals induce the peroxidation of PUFAs within the lipid membrane. This lethal process is physiologically countered by the GSH system, regulated by the enzyme GPX4, which coverts LOOH to LOH to maintain membrane integrity. Right panel (cuproptosis): copper-induced death occurs primarily within the mitochondrial matrix. Excess Cu^2+^ is reduced to Cu^+^ by the upstream regulator FDX1. The resulting Cu^+^ promotes the lipoylation and subsequent toxic aggregation of DLAT. This proteotoxic “clumping” (DLAT)_n_, combined with the destabilization and loss of Fe-S cluster proteins, leads to catastrophic mitochondrial metabolic failure. While both pathways share a reliance on metal-induced oxidative stress (highlighted by yellow arrows converging on the final execution of cell death), they represent fundamentally different modes of execution: lipid-driven membrane failure (ferroptosis) vs. protein-driven metabolic catastrophe (cuproptosis). Experimentally, these pathways are distinguished in nanomaterials-based systems by the specific detection of FDX1-dependent DLAT oligomerization versus GPX4-regulated lipid peroxidation.

Mechanistically, copper toxicity is inseparable from its redox activity; beyond proteotoxic aggregation, copper redox cycling independently contributes to the destabilization of Fe-S clusters, distinguishing this protein-driven catastrophe from lipid-driven membrane failure [17]. The comparative biochemical frameworks of these two pathways—specifically the contrast between lipid-driven membrane failure in ferroptosis and protein-driven metabolic catastrophe in cuproptosis—are illustrated in Figure 1.

The clinical relevance of these pathways is growing rapidly. In cancer treatment, inducing ferroptosis and cuproptosis offers powerful strategies to overcome drug resistance, particularly apoptosis-resistant malignancies [13]. However, cancer cells possess active homeostatic mechanisms, such as overexpression of the copper-transporting ATPase 1 (ATP7A) or elevated levels of GSH, which can restrict therapeutic efficacy [19]. GSH serves a dual-protective role: it is the essential electron donor for the anti-ferroptotic enzyme GPX4 and also acts as a high-affinity endogenous copper chelator [20]. Conversely, in neurodegeneration, ferroptosis is implicated in Alzheimer’s disease (AD) [21], Parkinson’s disease (PD) [22], stroke [23], amyotrophic lateral sclerosis (ALS) [24], and multiple sclerosis (MS) [25]. In PD, focal iron accumulation is associated with dopaminergic neurodegeneration, and studies suggest that methamphetamine (METH) neurodegeneration might be mediated by iron-dependent ferroptosis in the nigrostriatal system [26]. Furthermore, dysregulation of copper-iron metabolism is evident in diseases like MS, where a copper chelator (cuprizone) induces demyelination by disrupting systemic iron homeostasis and triggering oxidative damage in oligodendrocytes [27].

**Figure 2:**
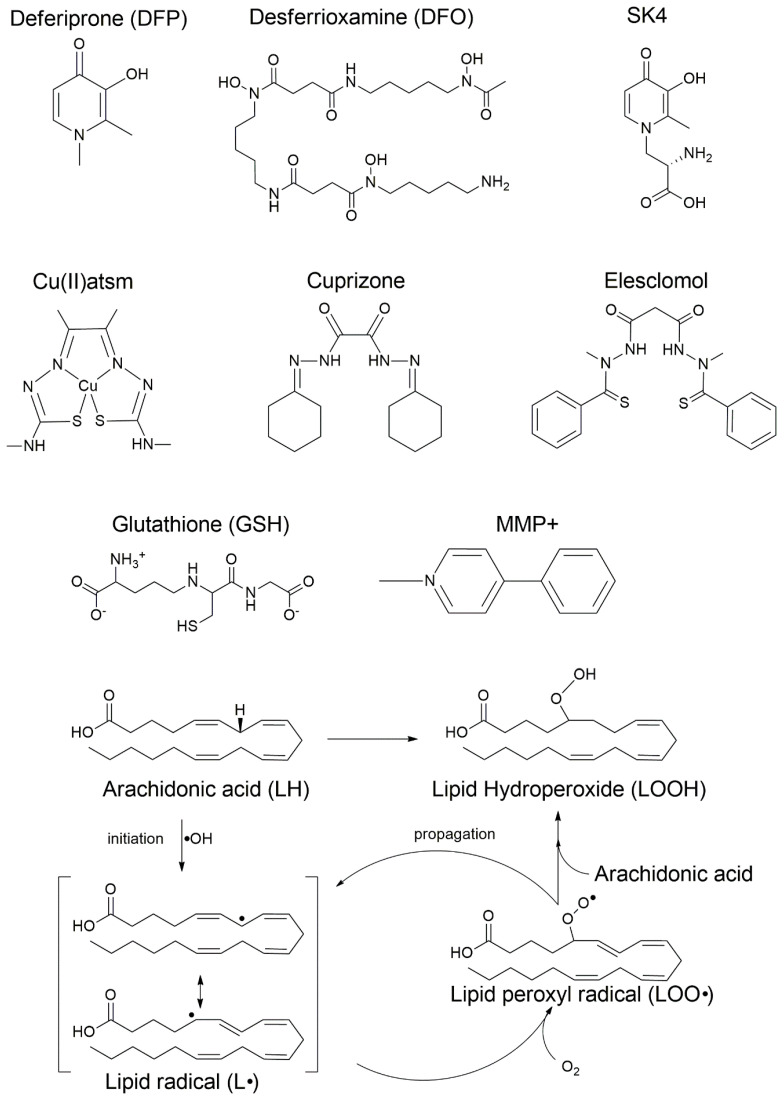
Chemical structures of key therapeutic modulators and the LPO pathway. The figure displays the central regulatory and therapeutic molecules involved in the copper-iron-ferroptosis nexus. Top panel: deferoxamine (DFO), deferiprone (DFP), and SK4, high-affinity iron chelators used to sequester Fe^2+^ and inhibit Fenton-driven initiation; diacetylbis(N_4_-methylthiosemicarbazonato)copper(II) (Cu^II^(atsm)), a copper-based complex with potent anti-ferroptotic activity; Cuprizone, a copper chelator utilized in experimental models of demyelination to induce metal-homeostasis disruption, and elesclomol, a novel copper transporter that binds extracellular Cu(II) and releases Cu(I) into the cell; GSH, an essential endogenous antioxidant, and MMP^+^, an apoptotic inducer and neurotoxin. Bottom panel: mechanism of LPO and lipid priming. The radical chain reaction is illustrated through four stages of a substrate-specific process: (1) Priming: the enzymatic activation of polyunsaturated fatty acids (PUFAs), specifically arachidonic acid (LH), for membrane incorporation via the ACSL4-LPCAT3 axis, which generates the required PUFA-PE context; (2) Initiation: the formation of the carbon-centered lipid radical (L•) via iron-catalyzed hydroxyl radical (•OH) initiation; (3) Oxygenation: the generation of the lipid peroxyl radical (LOO•); and (4) Propagation: the primary toxic product, LOOH, is formed as LOO• reacts with adjacent PUFA-PEs in a self-amplifying cycle. The “vicious cycle” concludes in membrane rupture when these specific phospholipid substrates are exhausted or their repair mechanisms fail.

A prominent approach to modulating these cell death pathways involves the use of high-affinity metal chelators and related agents. For instance, iron chelators like DFO and DFP can block ferroptosis in both cancer and neurodegeneration contexts, providing a strategy for neuroprotection [28,29]. Similarly, the anti-ferroptotic activity of copper compounds like Cu^II^(atsm) suggests their potential as neuroprotective agents via the stabilization of redox homeostasis [30]. In oncology, innovative nanoplatforms incorporating bimetallic Fe/Cu frameworks or ionophores are being developed to overcome delivery barriers and exploit the synergistic killing effects of co-activating ferroptosis and cuproptosis [31,32]. The chemical structures of these key therapeutic modulators, alongside the “vicious cycle” of the LPO pathway they aim to interrupt, are detailed in Figure 2. This complexity underscores the critical therapeutic potential residing in the precise regulation of metal ion availability and downstream effectors like GPX4.

## 2. Comparative Therapeutic Paradigms: Metal-Driven RCD as a Weapon vs. a Target

This article systematically compares and synthesizes evidence regarding the application of metal chelators and related agents to achieve two fundamentally opposing therapeutic outcomes in major human pathologies: oncology and neurodegeneration. In the context of cancer treatment, the objective is the deliberate induction of RCD, specifically leveraging metal toxicity, primarily through enhancing ferroptosis and cuproptosis, to eliminate malignant cells [20,33,34,35,36,37]. Conversely, in neurodegenerative disorders, the corresponding goal is the inhibition of RCD (largely ferroptosis) by chelating and sequestering toxic accumulations of biometals, predominantly Fe and Cu, thereby conferring neuroprotection to vulnerable neural tissues [26,36,38,39]. The key differences between these two therapeutic paradigms are summarized in Table 2.

The susceptibility to these pathways is fundamentally governed by cell-type specific regulatory mechanisms. Malignant cells often exploit ferritinophagy (nuclear receptor coactivator 4 (NCOA4)-mediated ferritin degradation) to increase labile iron for proliferation, while simultaneously utilizing transcriptional regulation of transporters like TfR1 to satisfy “iron addiction”. Conversely, in the central nervous system (CNS), oligodendrocytes are uniquely vulnerable to ferroptosis due to high iron requirements for myelin synthesis and a disproportionately low redox buffering capacity [40]. Neurons maintain homeostasis through localized mitophagy and immune-dependent control, where neuroinflammatory signals can either trigger or suppress ferroptotic cascades [41]. Integrating these intrinsic biological constraints with nanoplatform design is essential for achieving therapeutic precision across different cellular environments.

This section offers several novelty highlights within this comparative framework:

(1) Dual comparative focus: We directly juxtapose the distinct mechanistic requirements—RCD induction versus RCD inhibition—and their associated therapeutic agents and delivery systems. In oncology, the strategy capitalizes on tumor cell vulnerabilities, such as “iron addiction” and high metabolic activity, which make them susceptible to Fe- and Cu-mediated oxidative stress [30,33,35,42]. In neurodegeneration, the goal is reversal of pathology, addressing iron accumulation implicated in processes like dopaminergic neurodegeneration in PD and oligodendrocyte loss in the context of MS [26,36,38,39].
(2) Integration of cuproptosis and the iron-copper nexus: The analysis systematically integrates the recently discovered cuproptosis alongside ferroptosis, analyzing the pivotal and synergistic interplay (or crosstalk) between Fe and Cu metabolism in inducing cell death. This crosstalk is a primary therapeutic target. In this mechanism, the reduction of copper ions (Cu^2+^ to Cu^+^) drives cuproptosis. Simultaneously, this process depletes cellular GSH, which critically inactivates the key ferroptosis regulator GPX4 [20,31,33,34,43,44,45,46].
(3) Focus on advanced agents: We emphasize the deployment of advanced agents and sophisticated delivery systems, including advanced nanoplatforms (such as bimetallic Fe/Cu MOFs) and targeted nano-ionophores, designed to overcome biological barriers and host homeostatic resistance mechanisms. This includes strategies to deliberately inhibit copper efflux transporters like ATP7A and iron exporters like FPN1 to promote fatal intracellular metal accumulation in cancer cells [31,33,34,46]. Conversely, in neurodegeneration, synthetic iron chelators like SK4 are specifically developed to penetrate the BBB via large neutral amino acid transporters (LAT1) for targeted neuroprotective efficacy. While xCT (the active subunit of System $x_{c}^{-}$) remains the primary regulator of GSH synthesis and ferroptosis defense, SK4 utilizes a different pathway for CNS entry. This agent leverages the LAT1 transporter, which is typically responsible for the uptake of large neutral amino acids. By hijacking this mechanism, SK4 ensures successful drug delivery across the blood–brain barrier [38,47,48].

**Figure 3:**
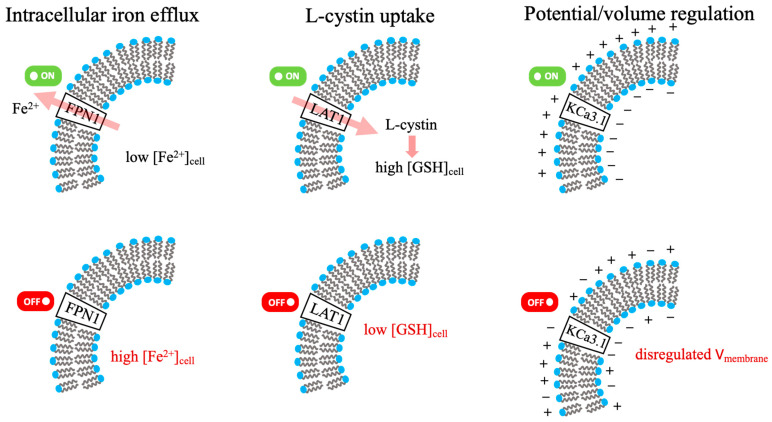
FPN1, LAT1, KCa3.1: membrane regulation of cellular Iron, GSH, and electrophysiology. (Green/red indicators denote activated/inhibited states; pink arrows represent the direction of substrate flux).

The specific roles of FPN1, LAT1, and the KCa3.1 ion channel in the membrane regulation of cellular iron, GSH, and electrophysiology are illustrated in Figure 3 [49,50,51]. The roles of these central regulatory proteins, transporters, and ion channels in maintaining metal and redox homeostasis are further detailed in Table 3.

In essence, this section examines how metal dyshomeostasis is simultaneously treated as both a therapeutic target (requiring protection in neurodegeneration) and a therapeutic weapon (requiring cell death induction in cancer), while elucidating the metabolic complexity stemming from the combined biological effects of Fe and Cu [31,33,34,35,36].

## 3. Cancer: Synergistic Induction of Regulated Cell Death

The efficacy of chemotherapy in treating many aggressive cancers is frequently limited by the tumor cells’ ability to resist apoptosis [33]. Consequently, there has been a significant emergence of strategies focusing on disrupting metal ion homeostasis to induce distinct modalities of RCD, namely ferroptosis and cuproptosis. The co-activation of these Fe and Cu driven cell death pathways offers a promising strategy to overcome drug resistance and enhance therapeutic outcomes [31,44].

### 3.1. Dual-Activation Strategies via Advanced Nanoplatforms

The nanotherapeutic induction of dual cell death relies on the fundamental biochemical pathways established in Section 1 and Figure 1. Specifically, instead of re-detailing the GSH/GPX4/LPO cascade, this section examines how advanced platforms manipulate these established vulnerabilities to overcome tumor resistance. Achieving synergistic cuproptosis and ferroptosis often requires advanced nanoparticle (NP) systems capable of overcoming biological delivery barriers, evading cellular homeostasis mechanisms, and promoting the necessary redox chaos within the tumor microenvironment (TME).

#### 3.1.1. Bimetallic Frameworks (Fe & Cu)

**Figure 4:**
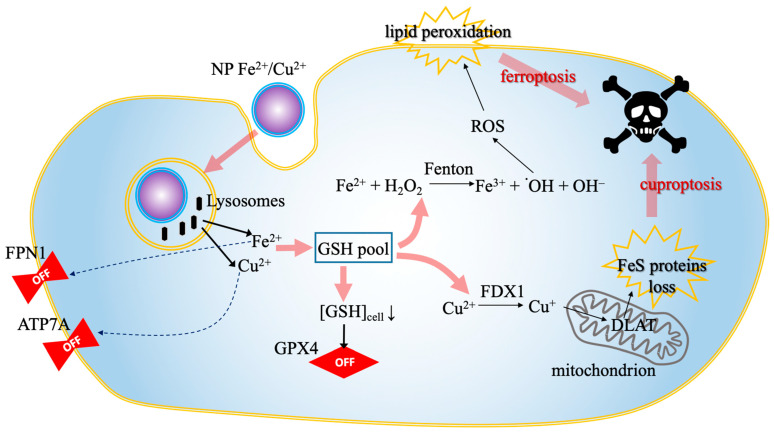
Nanomaterial-mediated dual RCD induction. The schematic illustrates the synergistic intracellular cascade triggered by bimetallic nanoplatforms. The process proceeds in four stages: (1) the nanoparticle is internalized and degraded within lysosomes, releasing bioactive Fe^2+^ and Cu^2+^ ions (black arrows); (2) these ions downregulate (dotted arrows) the efflux transporters ATP7A and FPN1, effectively trapping the metal load inside the cell; (3) the surge in metal ions depletes the GSH pool (pink arrows), which simultaneously inactivates the anti-ferroptotic enzyme GPX4 and facilitates the reduction of Cu^2+^ to Cu^+^ via FDX1; (4) this metabolic collapse culminates (pink arrows) in dual cell death: Cu^+^ drives mitochondrial Fe-S cluster protein loss (cuproptosis), while the iron-driven Fenton reaction generates ROS, leading to lethal lipid peroxidation (ferroptosis). Abbreviations: ATP7A, copper-transporting ATPase 1; Cu^+^, monovalent copper; Cu^2+^, divalent copper; FDX1, ferredoxin 1; Fe^2+^, ferrous iron; Fe-S, iron-sulfur cluster; FPN1, ferroportin 1; GPX4, glutathione peroxidase 4; GSH, glutathione; NP, nanoparticle; RCD, regulated cell death; ROS, reactive oxygen species.

Bimetallic nanoplatforms integrate both Fe and Cu ions—the key initiators of ferroptosis and cuproptosis—into a single carrier, thereby maximizing the synergistic effect against malignant cells [34,45]. Examples include copper/iron hybrid hollow amorphous MOFs (HaMOF) [33], copper/iron bimetal gallic acid (CuFeGA) and metal-phenolic networks (MPNs) [52], and copper-doped hollow Prussian blue (PB) nanozymes [34]. These systems are designed to amplify cellular oxidative stress and disrupt metal metabolism simultaneously [33]. The integrated mechanism of nanomaterial-mediated dual RCD induction, characterized by the concurrent depletion of GSH and the trapping of metal ions to amplify both ferroptosis and cuproptosis, is illustrated in Figure 4.

i. Ferroptosis pathway: Bimetallic frameworks primarily enhance ferroptosis by disrupting the cellular antioxidant defense system and boosting the production of lethal reactive oxygen species (ROS) [34]. The initial step often involves the Fe and Cu ions acting as dual GSH depletors. For example, CuFeGA MPNs rely on two procedures of GSH consumption, releasing oxidized metal ions (Fe^3+^ and Cu^2+^), which are then rapidly reduced by GSH (consuming the GSH and generating Fe^2+^ and Cu^+^) to initiate the first phase of GSH depletion. Subsequently, the released Gallic acid (GA) further consumes the residual GSH. This robust GSH depletion subsequently inactivates the master ferroptosis regulator, GPX4, preventing it from converting toxic LOOH into harmless LOH. Concurrently, the released Fe^2+^ and Cu^+^ catalyze Fenton and Fenton-like reactions, reacting with endogenous hydrogen peroxide (H_2_O_2_) to generate highly toxic hydroxyl radicals. This surge in ROS and consequent oxidative stress leads to the accumulation of LOOH, ultimately initiating augmented ferroptosis [34,52,53]. Furthermore, some nano-systems like the HaMOF induce metabolic disorders that specifically downregulate the copper transporter ATP7A and contribute to high iron retention, which supports the Fenton reaction and enduring ferroptosis [33].

ii. Cuproptosis pathway: Cuproptosis induction via bimetallic nanoplatforms relies heavily on accumulating sufficient intracellular Cu^+^ and circumventing the protective effects of GSH. The initial release of Cu^+^ (and its generation via the reduction of Cu^2+^) is critical for inducing cuproptosis. This accumulation of excessive Cu^+^ in the resulting GSH-depleted environment causes the aggregation of lipoylated proteins, notably DLAT. The aggregation of DLAT interferes with the mitochondrial Krebs cycle and leads to the loss of iron-sulfur (Fe-S) cluster proteins, culminating in proteotoxic stress and cuproptosis [34,52]. Mechanistically, the synergistic effect often relies on feedback loops: HaMOF treatment causes mitochondrial damage and subsequently disrupts adenosine triphosphate (ATP) production. This reduction in cellular energy suppresses the expression of copper exporters, such as ATP7A, thereby enhancing copper ion retention in the cytoplasm and further promoting cuproptosis [33,44].

#### 3.1.2. Targeted Nano-Ionophores

Targeted strategies employ sophisticated carriers to deliver metal ions or small molecules capable of disrupting cellular copper homeostasis, often specifically inhibiting efflux pathways. One strategy involves CRISPR-Cas9 ribonucleoprotein (RNP) nanocarriers, specifically the RNP@Cu_2_O@SPF platform, which utilizes a silica-polydopamine-folic acid (SPF) shell to facilitate targeted delivery [46]. These nanoconstructs are engineered to transport the RNP complex—comprising the Cas9 endonuclease and a single-guide RNA targeting the ATP7A gene (sgATP7A)—to achieve the site-specific gene silencing of the copper transporter ATP7A [46,54]. The degradation of the nanocarrier in the GSH-rich, weak acidic tumor environment releases RNP and Cu^+^ ions. By inhibiting ATP7A, this system ensures sustained high copper levels in the cytoplasmic fluid, which subsequently enhances the efficacy of cuproptosis, ferroptosis, and associated chemodynamic therapy (CDT) [18,46].

A similar concept is implemented using the CussOMEp nanozyme system, which integrates a copper-based nanovector with the copper-transporter inhibitor omeprazole. In this architecture, omeprazole serves to selectively block the ATP7A exporter, driving significant intracellular Cu^2+^ accumulation and escalating oxidative stress. This copper overload promotes the oligomerization of DLAT proteins, a critical step in the induction of cuproptosis. Furthermore, the copper-based nanovector itself exhibits multi-enzymatic mimicry—including peroxidase, catalase, and glutathione peroxidase-like activities—enabling the catalytic conversion of H_2_O_2_ into hydroxyl radicals and oxygen. Simultaneously, the platform depletes intracellular GSH, creating a metabolic environment that robustly synergizes cuproptosis with enhanced ferroptosis [55,56].

Furthermore, carrier-free metal-polyphenolic nanocomplexes (T-T@Cu) utilize tannic acid for mitochondrial targeting. This nanoplatform can induce mild photothermal-boosted ferroptosis, which results in decreased cellular ATP levels. This energy depletion subsequently downregulates the expression and activity of copper efflux proteins (ATP7A/7B), thereby disrupting copper homeostasis and significantly exacerbating cuproptosis in tumor cells [57].

### 3.2. Small-Molecule Synergy and Therapeutic Outcomes in Cancer

#### 3.2.1. Small-Molecule Dual-Activation

The efficacy of anti-cancer treatments has been profoundly augmented by strategies exploiting the intricate crosstalk between distinct forms of RCD, particularly the iron-dependent ferroptosis and the copper-dependent cuproptosis. This synergistic potential is frequently realized through the deployment of specialized small molecules and nanoplatforms designed to disrupt metal homeostasis and cellular redox balance concurrently [31,33,34,44]. A central nexus in this synergy is the cellular antioxidant GSH, which acts as a co-inhibitor for both ferroptosis (via the GPX4 pathway) and cuproptosis (by chelating copper ions). Consequently, inducing a state of dual GSH depletion is an established strategy to sensitize tumor cells to both pathways [34,44,52].

Nanoplatforms are engineered specifically to exploit these shared metabolic vulnerabilities. For instance, CuFeGA MPNs enable a phased depletion of GSH: upon exposure to the high-GSH TME, the networks dissociate to release GA, Fe^2+^, and Cu^+^, followed by secondary GSH consumption mediated by the released GA. The resulting surge in bimetallic ions drives intense oxidative stress via Fenton and Fenton-like reactions, precipitating LPO and the downregulation of GPX4 to trigger ferroptosis. Simultaneously, the elevated Cu^+^ levels induce the oligomerization of lipoylated DLAT, thereby activating the cuproptosis pathway [52]. A similar synergistic effect is achieved using bimetallic Fe-Cu MOFs, which utilize the coordinated action of iron and copper to escalate cellular damage beyond the thresholds achievable by single-agent therapies. This dual-metal strategy ensures that the induction of ferroptosis and cuproptosis is mutually reinforcing, effectively overcoming the robust antioxidant defenses typical of malignant cells [31].

Beyond direct metal delivery, small molecules targeting fundamental metabolic regulators can achieve potent synergistic anti-cancer effects. Ferroptosis inducers, such as sorafenib and erastin, significantly potentiate copper ionophore-induced cuproptosis in hepatocellular carcinoma cells. This synergy is driven by two complementary mechanisms: first, these inducers deplete the intracellular GSH pool by inhibiting the cystine/glutamate antiporter (System $x_{c}^{-}$). Second, they augment the cuproptosis pathway by stabilizing FDX1 through the suppression of mitochondrial matrix-related proteases, thereby preserving the protein lipoylation essential for copper-induced cell death [58,59].

Furthermore, nanotherapeutic strategies specifically designed to overcome copper efflux mechanisms have been developed. Elesclomol, a copper chelator, induces copper overload and subsequent ferroptosis in colorectal cancer (CRC) cells by promoting the ubiquitin-mediated degradation of the copper efflux transporter ATP7A, leading to copper retention in mitochondria and subsequent ROS accumulation [60]. In a similar vein, the mild photothermal-boosted ferroptosis approach (T−T@Cu), a carrier-free metal-polyphenolic nanocomplex based on tannic acid and copper, exploits the resultant depletion of cellular ATP to disrupt the function and expression of copper efflux proteins, such as ATP7A/7B, thereby maintaining high intracellular Cu levels necessary for robust cuproptosis induction [44,57].

Importantly, this synergy represents a causal hierarchy rather than mere metabolic co-occurrence. Initial iron-driven lipid peroxidation inflicts catastrophic mitochondrial damage that depletes intracellular ATP stores. Because the primary copper exporters (ATP7A/B) are P-type ATPases, this bioenergetic “power outage” effectively locks copper within the cytosol, transforming a sublethal copper load into a toxic trigger for secondary cuproptosis via DLAT aggregation [19]. In this contingent cascade, ferroptosis acts as the metabolic initiator that disables the cell’s ability to clear the copper load, while copper-mediated glutathione depletion simultaneously lowers the kinetic barrier for ferroptotic propagation. This mutually reinforcing feedback loop ensures that the activation of one pathway creates the obligatory metabolic prerequisites for the other [3,5], circumventing the redundant antioxidant defenses typical of resistant malignancies. These findings underscore the mutually reinforcing therapeutic relationship, where copper and iron homeostasis disruption pathways are utilized to amplify ROS-driven cellular destruction [34,44].

**Figure 5:**
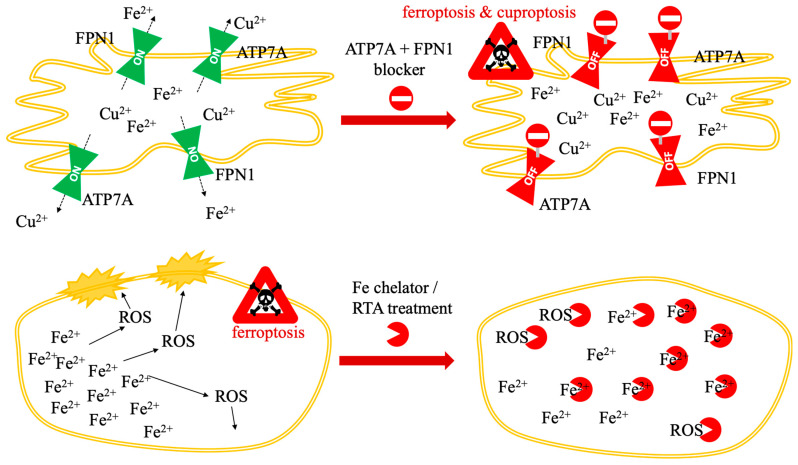
Targeted modulation of metal homeostasis: strategies for killing (Fe/Cu accumulation) and protection (Fe chelation/ROS neutralization).

The overarching principles of these metal-centric interventions are summarized in Figure 5, which illustrates the targeted modulation of metal homeostasis: strategies for killing via Fe/Cu accumulation in cancer versus strategies for protection via Fe chelation and ROS neutralization in other pathological states.

#### 3.2.2. Ferroptosis-Only Mechanisms

Ferroptosis, defined as an iron-dependent form of cell death driven by LOOH accumulation, can be specifically manipulated using various small molecules and engineered nanoplatforms that regulate iron availability and mitigate oxidative stress. Targeting iron metabolism directly through chelation remains the foundational strategy for preventing ferroptosis [26,36,39]. Traditional iron chelators like DFO are highly effective at blocking ferroptosis. The clinical relevance of this approach is validated by studies showing that the orally available chelator DFP decreased iron content in the substantia nigra of PD patients, which is associated with slower disease progression [38,39]. Extending this strategy, the small molecule SK4 was rationally designed to incorporate structural elements facilitating translocation across the BBB via the LAT1 transporter. Detailed physicochemical analysis confirmed that SK4 exhibited a stronger complex formation affinity toward Fe^3+^ than DFP, especially within the higher pH milieu characteristic of mitochondria, providing superior protection against Fe^2+^ and MPP^+^ (1-methyl-4-phenylpyridinium)-induced neurodegeneration [38]. Furthermore, the peptide thymosin β4 has been identified as an endogenous iron chelator capable of binding both Fe^2+^ and Fe^3+^ ions in four distinct regions of the peptide, consequently inhibiting erastin- and glutamate-induced ferroptosis [35].

Alternatively, ferroptosis can be averted through radical scavenging that halts LPO. Potent inhibitors like Ferrostatin-1 (Fer-1) and Liproxstatin-1 (Lip-1) function as radical trapping antioxidants (RTAs) [36]. Research into metal complexes identified that the copper complex Cu^II^(atsm), currently in clinical trials for neurodegenerative diseases, prevents ferroptotic cell death in neuronal models with a potency comparable to Lip-1. This antiferroptotic activity is attributed to its function as a lipid RTA, quenching lipid peroxyl radicals without modulating the oxidation state of iron species. Intriguingly, the analogous nickel complex Ni^II^(atsm) showed comparable protective effects, suggesting that the underlying mechanism lies primarily in the delocalized conjugated conformation of the ligand rather than merely the copper ion itself [30].

Ferroptosis is also modulated by non-iron metals. Studies have demonstrated that zinc significantly affects ferroptosis sensitivity: while zinc chelators confer resistance, the addition of exogenous ZnCl_2_ promotes cell death, even counteracting the protective effect of the iron chelator DFO. This process is regulated by the ZIP7 transporter, which facilitates the release of Zn^2+^ from the endoplasmic reticulum into the cytosol—a flux essential for maintaining organelle homeostasis and modulating ferroptotic pathways [61]. This is exemplified by ZnO nanoparticles, which utilize ion-leaking properties to disrupt intracellular iron uptake and storage mechanisms. This zinc-mediated disruption triggers a p53-dependent downregulation of GPX4 and GSH depletion, proving that exogenous iron is not a prerequisite for ferroptotic induction if endogenous metal homeostasis is sufficiently destabilized [62].

Finally, nanomaterials offer diverse pathways to inhibit ferroptosis. Carbon dot nanozymes protect cells by chelating Fe^2+^ via surface functional groups (carbonyl, hydroxyl, carboxyl) to inhibit the Fenton reaction, while simultaneously scavenging highly ROS [63]. Similarly, polydopamine (PDA) NPs mitigate intervertebral disc degeneration (IVDD)-associated ferroptosis through three distinct mechanisms: ROS scavenging, Fe^2+^ chelation, and, notably, suppression of the ubiquitin-mediated degradation of the anti-ferroptotic enzyme GPX4 [64].

#### 3.2.3. Therapeutic Outcomes

The fundamental research into small-molecule and nano-mediated regulation of ferroptosis and cuproptosis pathways has translated into promising therapeutic strategies across oncology, neurodegeneration, and acute tissue injury.

In oncology, the focus is largely on utilizing the intrinsic synergy between Fe and Cu pathways to enhance tumor cell destruction and augment immune response activation. An actively targeted magneto-liposome (T-LMD), co-encapsulated with oleic acid-coated iron oxide NPs and doxorubicin, demonstrated superior anti-tumor efficacy in human triple negative breast carcinoma (TNBC) cells, primarily by activating ferroptosis through enhanced LPO and ROS generation [65]. Moreover, advanced combination nanotherapies leveraging metal-mediated cell death pathways significantly potentiate immunotherapy. For instance, core–shell nanoparticles combining copper and erastin strongly inhibits tumor growth in murine colon and breast cancer studies. When coupled with immune checkpoint blockade (ICB), the combination therapy effectively synergized T cell proliferation and reinvigoration, leading to potent tumor regression and prevention of metastasis by inducing robust immunogenic cell death [37,66]. This strategy is further validated by the development of bimetallic Fe-Cu MOFs, which have demonstrated the ability to effectively eliminate solid tumors and significantly extend survival in tumor-bearing mice through the co-activation of cuproptosis and ferroptosis [31]. Similarly, specialized copper-based nanozymes (such as CussOMEp), engineered to enhance intracellular Cu^2+^ accumulation and synergize Fe/Cu pathways, have shown potent inhibition of tumor metastasis when integrated with targeted immunotherapy [55].

In neurodegenerative disorders, high iron deposition in the CNS is a characteristic finding in conditions like PD, making iron-dependent ferroptosis a promising therapeutic target [26,36,38]. The specialized iron chelator SK4, capable of penetrating the BBB, provided robust neuroprotection in experimental models of PD by mitigating MPP^+^ and Fe^2+^-induced toxicity [38]. The iron chelator DFO and the ferroptosis inhibitor Lip-1 also successfully reduced intracellular oxidative stress and protected dopaminergic neurons in vitro from MPP^+^ damage [39]. In addition, the complex Cu^II^(atsm) is currently being investigated in clinical trials for ALS and PD due to its potent, brain-penetrant antiferroptotic activity, acting as a lipid RTA [30].

Ferroptosis is also implicated in acute tissue damage such as hepatic ischemia–reperfusion (I/R) injury, where donor iron overload is identified as an independent risk factor for liver damage following liver transplantation. In murine models, I/R injury induced LPO and hallmark ferroptotic signaling, effects that were significantly attenuated by the specific ferroptosis inhibitor Fer-1 or the iron chelator DFO. Iron overload specifically exacerbated I/R injury, confirming ferroptosis involvement [67]. Inhibiting ferroptosis with Fer-1 also suppressed subsequent inflammatory responses, suggesting that ferroptosis is an upstream trigger for necroinflammation in hepatic I/R injury [68]. Beyond liver injury, carbon dot nanozymes have demonstrated success in alleviating renal I/R injury by chelating Fe^2+^ and scavenging ROS to prevent ferroptosis [63]. Similarly, PDA NPs efficiently mitigated IVDD in rats by directly targeting ferroptosis pathways, including inhibiting GPX4 ubiquitination and reducing iron accumulation, thereby offering a novel therapeutic strategy for musculoskeletal disorders [64].

## 4. Neurodegeneration: Metal Chelators as Neuroprotective Agents

### 4.1. Iron Chelation for Neuroprotection and Acute Injury Mitigation

The dysregulation of metal ion homeostasis, particularly the abnormal accumulation of iron in the CNS, is a critical factor implicated in the pathology of numerous neurological disorders such as PD, AD, ALS, stroke, and MS. Ferroptosis, an iron-dependent form of non-apoptotic cell death characterized by catastrophic LPO, is considered a significant mechanism underlying neuronal loss in these conditions [26,30,36,69]. Therefore, targeting iron overload or inhibiting the ferroptosis pathway using metal chelators represents a compelling therapeutic strategy for neuroprotection [36,38]. Targeting iron-dependent ferroptosis in neural tissues utilizes the same regulatory machinery described previously; however, the therapeutic objective shifts from the induction of LPO to its prevention through site-specific chelation and antioxidant stabilization [21,70]. Importantly, while ferroptosis is a potent driver of neuronal loss, it does not operate in isolation; rather, it represents a convergent terminal pathway integrated with mitochondrial dysfunction, glutamate-induced excitotoxicity, and impaired mitophagy, where iron-dependent lipid peroxidation acts as the biochemical tipping point once parallel homeostatic mechanisms are overwhelmed [71].

#### 4.1.1. Classic Chelators

The fundamental approach to blocking ferroptosis relies on the application of iron chelators, notably DFO. DFO inhibits ferroptosis and associated neurodegeneration in various preclinical systems by sequestering excess iron, thereby mitigating iron-induced oxidative damage and preventing the initiation of the Fenton reaction [36,38,39,72].

In the Parkinsonian paradigm, the neurotoxicant MPP^+^, a common mimetic used to induce dopaminergic neuronal toxicity [73,74], triggers ferroptosis characterized by mitochondrial shrinkage and a marked reduction in the expression of the essential anti-ferroptotic protein GPX4. Treatment with the iron chelator DFO effectively protects these neurons in vitro by reducing intracellular oxidative stress and rescuing mitochondrial function, while simultaneously upregulating GPX4 and ferritin heavy-chain expression, indicating that DFO specifically inhibits the ferroptosis signaling pathway activated by MPP^+^ injury. Furthermore, DFO has demonstrated neuroprotective effects comparable to the canonical ferroptosis inhibitor Lip-1, suggesting that its efficacy stems primarily from the sequestration of the intracellular free ferrous iron pool, the primary driver of lipid radical generation [39].

In studies of chronic neurodegeneration, clinical trials assessing the orally available iron chelator DFP have shown varied but promising results in PD patients [75,76], with early-stage patients exhibiting decreased substantia nigra iron content and slowed disease progression associated with improved motor scores [38]. In mice subjected to chronic METH exposure, DFP effectively alleviated nigrostriatal iron deposition, LPO, and dopaminergic cell death, directly mitigating ferroptosis induced by chronic METH exposure. DFP treatment also attenuated the reduction in GPX4 expression, stabilizing the cell’s endogenous defense against oxidative damage. Importantly, DFP selectively reduced iron levels without influencing copper, zinc, manganese, or magnesium concentrations in the substantia nigra, suggesting a specific binding preference for iron at the tested concentrations [26,77].

#### 4.1.2. Targeted Small Molecules

Beyond conventional iron chelators, advanced strategies focus on engineering small molecules capable of efficiently crossing the BBB and selectively targeting neuronal vulnerability [36,38].

**Figure 6:**
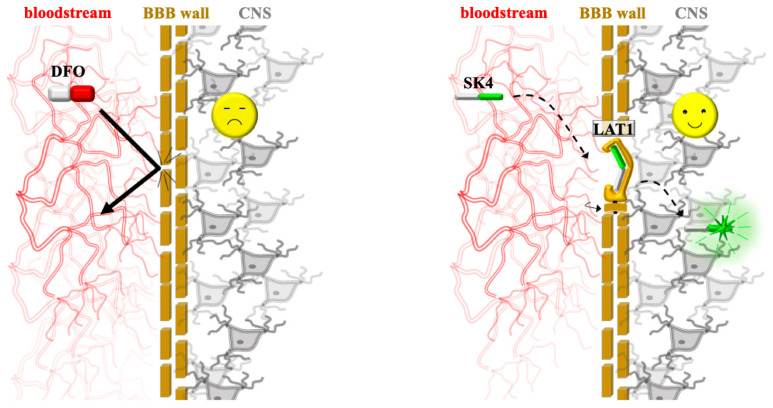
Targeted neuroprotective delivery via the BBB. (**Left**) Traditional chelation: large, hydrophilic agents like DFO are excluded by the BBB (bold arrow), failing to reach the CNS. (**Right**) Targeted delivery: the SK4 molecule is recognized by the LAT1 transporter (large neutral amino acid transporter 1). The drug utilizes (dotted arrows) the transporter’s revolving-door mechanism (represented by the rotating gate icon), where a conformational change in the protein physically carries the drug from the bloodstream into the brain tissue, enabling successful neuroprotection.

The hydroxypyridinone-based molecule SK4 exemplifies this design rationale, formulated to leverage the ubiquitous LAT1 for enhanced CNS penetration; by mimicking the structural features of L-DOPA—the primary clinical treatment for Parkinson’s disease, which also utilizes LAT1—SK4 achieves superior brain delivery compared to traditional chelators. Experimental validation confirmed SK4’s role as a LAT1 substrate by showing that it strongly competes with phenylalanine for uptake and significantly promotes phenylalanine export in a trans-stimulation assay—the definitive proof of the transporter’s obligatory exchange mechanism [38,78,79]. The contrast between traditional iron sequestration and this advanced transport method is illustrated in Figure 6, which highlights how the SK4 molecule bypasses the BBB—a barrier that typically excludes large, hydrophilic agents like DFO—by utilizing the LAT1 transporter’s conformational exchange to enter brain tissue.

In in vitro experiments utilizing human dopaminergic neurons, SK4 demonstrated robust neuroprotective properties against diverse neurotoxic agents [48,78]. In the context of ferroptosis induction, SK4 effectively rescued neuronal loss and maintained neurite integrity in cells treated with the inducer erastin, showing efficacy comparable to DFP. This protection was further validated in three-dimensional neuronal organoids challenged with erastin. Furthermore, SK4 fully protected these human neurons against MPP^+^-induced mitochondrial toxicity and broader oxidative stress, confirming its role as a specialized iron chelator in neurodegenerative contexts. SK4 also provided protection against damage induced by externally added ferrous sulfate, reinforcing its high affinity and selectivity for iron [38].

Regarding its mechanism of action, SK4 displays a strong Fe^3+^ complex formation capacity that is superior to DFP at higher pH values, such as those characteristic of the mitochondrial matrix; this suggests enhanced chelation potency in critical subcellular compartments. Notably, SK4 showed no direct interaction with major reactive oxygen or nitrogen species, establishing that its neuroprotective activity is mediated predominantly through site-specific iron chelation rather than non-specific antioxidant scavenging [38].

Another endogenously identified chelator, the peptide thymosin β4, chelates both Fe^2+^ and Fe^3+^ ions across four distinct binding regions, thereby inhibiting erastin- and glutamate-induced ferroptosis. By restoring normal cellular morphology in macrophage cell lines and mitigating damage linked to iron accumulation, this peptide functions as a “ferroptosis switcher” that regulates the transition between cell survival and death [35,80].

#### 4.1.3. Acute and Chronic Tissue Degeneration

Ferroptosis plays a critical role in both chronic neurodegenerative decline and acute tissue damage associated with I/R injury [36,67]. In acute hepatic I/R injury, which occurs during liver transplantation, iron accumulation is an independent risk factor for graft failure. This process involves programmed cell death and LPO, a cascade significantly attenuated by DFO pre-treatment. Furthermore, the specific ferroptosis inhibitor Fer-1, or the antioxidant α-tocopherol, effectively prevents hepatic damage and markers of ferroptotic signaling. Fer-1 also inhibits subsequent necroinflammation, suggesting that ferroptosis is an upstream trigger for the broader inflammatory response in hepatic I/R injury. This mechanism is further supported by the observation that dietary iron overload significantly exacerbates hepatic I/R injury and LPO, and this exacerbated damage remains sensitive to inhibition by Fer-1 [67,81].

In chronic CNS degeneration conditions such as MS, PD, and ALS, iron deposition in affected brain regions is a hallmark feature [70,71,82]. However, ferroptosis contributes to CNS pathology even in the absence of overt iron accumulation, often due to transient increases in bioactive iron coupled with reduced ferritin storage capacity—a phenomenon demonstrated in the cuprizone model of demyelination [36]. Regarding inhibition, the highly potent RTA Cu^II^(atsm), currently in clinical trials for ALS and PD, directly blocks ferroptotic neuronal death and LPO in vitro with nanomolar efficacy comparable to Lip-1. This antiferroptotic activity is attributed to Cu^II^(atsm) acting as a lipid RTA, independent of alterations to the oxidation state of intracellular iron. The analogous Ni^II^(atsm) complex exhibits comparable potency, suggesting that efficacy relies primarily on the conjugated conformation of the ligand as stabilized by the metal ion; this enables the ligand to serve as an H-atom donor to quench lipid peroxyl radicals. This mechanism, combined with favorable oral bioavailability and brain penetrance, highlights a promising avenue for disease-modifying therapy in ferroptosis-related neurological disorders [30].

### 4.2. Interplay with Copper Metabolism and the Iron-Copper Nexus

**Figure 7:**
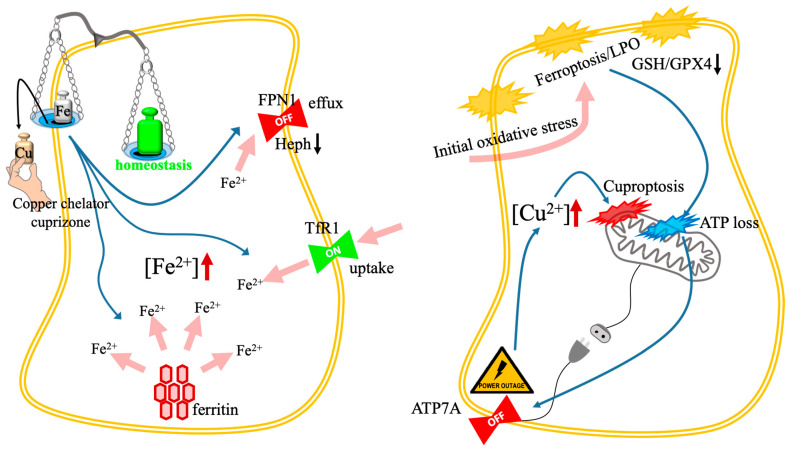
Toxic feedback loops and the iron-copper nexus. The schematic illustrates two distinct mechanisms of metal-dependent cell death resulting from the disruption of iron and copper interplay. (**Left**): The paradoxical iron trap. The cascade occurs as follows: (1) copper chelation (e.g., cuprizone; black arrow) depletes the copper cofactors required for hephaestin activity; (2) inactive hephaestin inhibits iron efflux via FPN1 (blue arrow); (3) concurrently, copper deficiency triggers the upregulation of TfR1 (blue arrow); (4) the resulting surge (pink arrows) in the intracellular LIP ([Fe^2+^]↑) drives lethal LPO and ferroptosis. (**Right**): The vicious cycle amplifier. Synergistic cell death is achieved in two stages: (1) initial oxidative stress (e.g., from Fe-Cu nanoplatforms; pink arrow) induces membrane LPO and GSH/GPX4 depletion; (2) mitochondrial damage leads to severe ATP loss (the “power outage”; blue arrow); (3) depletion of ATP inactivates the energy-dependent copper exporter ATP7A (blue arrow); (4) subsequent toxic copper accumulation ([Cu^2+^]↑) triggers secondary mitochondrial collapse via DLAT aggregation (cuproptosis), creating a self-amplifying feedback loop. Abbreviations: ATP7A, copper-transporting ATPase 1; DLAT, dihydrolipoamide S-acetyltransferase; FPN1, ferroportin 1; GPX4, glutathione peroxidase 4; GSH, glutathione; LIP, labile iron pool; LPO, lipid peroxidation; TfR1, transferrin receptor 1.

The intrinsic link between copper and iron metabolism is summarized in Figure 7, which illustrates how the disruption of one metal’s homeostasis can trigger toxic feedback loops—either through an “iron trap” mechanism (Panel A) or a “vicious cycle” of oxidative synergy (Panel B).

#### 4.2.1. Copper Chelator as Iron Driver

The metabolism of copper and iron ions is intrinsically linked, a phenomenon most clearly demonstrated by the unexpected induction of iron-dependent ferroptosis following copper chelation in neurodegenerative scenarios [83]. In the cuprizone mouse system, a widely used system to study demyelination relevant to MS, the copper chelator cuprizone triggers oligodendrocyte loss via ferroptosis. This toxicity arises because copper is an obligatory cofactor for multicopper ferroxidases, such as ceruloplasmin and hephaestin, which are required for efficient cellular iron efflux [36,84].

Chelation of copper disrupts this intricate system in oligodendrocytes, resulting in heightened intracellular iron load through multiple pathways [36,85]. Specifically, cuprizone reduces the functional activity of the oligodendrocyte-specific multicopper oxidase hephaestin, which impairs the oxidation of Fe^2+^ to Fe^3+^ required for export via ferroportin, thus inhibiting iron efflux. Simultaneously, copper chelation upregulates the transferrin receptor 1, increasing iron uptake. Critically, the process accelerates the expression of the ferritin cargo receptor, leading to ferritinophagy—the targeted autophagic degradation of the iron storage protein ferritin. This releases bioactive Fe^2+^ from storage into the LIP. The resulting surge of unbound Fe^2+^ generates massive ROS via the Fenton reaction, overwhelming the depleted antioxidant defenses (GSH and GPX4 are reduced), thereby driving lethal LPO and initiating ferroptosis [36,86].

#### 4.2.2. Copper Chelator as Inhibitor (Non-Cancer Context)

Conversely, certain copper-containing molecules function actively as inhibitors of ferroptosis, particularly in neuronal protection, through a mechanism independent of iron chelation. The complex Cu^II^(atsm) ameliorates neurodegeneration and delays disease progression in preclinical studies of ALS and PD, suggesting its mechanism involves blocking ferroptosis. Mechanistic investigation confirms that Cu^II^(atsm) acts as a potent RTA, effectively blocking ferroptotic neuronal death and inhibiting LPO in vitro with nanomolar efficacy comparable to Lip-1 [30].

The specific antiferroptotic activity of Cu^II^(atsm) and its analogous Ni^II^(atsm) is derived from the coordinated ligand structure, rather than the metal ion itself promoting ROS scavenging [30,87]. The complex inhibits LPO without altering the oxidation state of iron species. The metal ion -Cu(II) or Ni(II)- facilitates the necessary double deprotonation of the ligand, forming a resonance-stabilized conjugated conformation where the NH-CH_3_ functional groups act as H-atom donors to quench chain-carrying lipid peroxyl radicals. This structural requirement is critical: modifications that replace these essential NH-CH_3_ groups markedly attenuate the complex’s ability to prevent ferroptosis. Conversely, simply increasing intracellular copper concentrations via ionic copper (CuSO_4_) or non-protective copper ionophores fails to prevent ferroptosis. This demonstrates that the therapeutic mechanism is specific to the complex acting as an RTA and is not achieved merely by elevating cellular copper levels [30].

#### 4.2.3. The Iron-Copper Nexus

In oncology, the co-delivery of iron and copper ions has emerged as a superior strategy to synergistically induce cell death, overcoming the resistance posed by redundant survival pathways. This synergy is rooted in the mutually reinforcing disruption of cellular redox homeostasis and metal efflux mechanisms. Mechanistically, therapeutic effectors, often delivered via bimetallic nanoplatforms such as Fe-Cu MPNs or specialized hollow PB nanozymes, leverage the catalytic activity of both metals. The platforms release Fe^2+^ and Cu^+^ in the acidic, GSH-rich TME, facilitating Fenton or Fenton-like reactions that generate highly toxic hydroxyl radicals and ensuing LPO. Critically, this process simultaneously targets the central cellular defense system by rapidly depleting GSH, which serves both as a cofactor for GPX4 and a chelator for Cu^+^ [31,33,34,44,45,52].

Specific strategies refine this dual attack through integrated metabolic interference. First, dual GSH depletion and triple synergy is achieved with nanoplatforms like CuFeGA MPNs that exploit a novel dual GSH consumption approach: initial exposure to TME triggers the dissociation of the MPNs, releasing GA, Fe^2+^, and Cu^+^ for primary GSH consumption, followed by a second phase where the liberated GA further accelerates the depletion of remaining GSH. This profound redox collapse leads to the simultaneous activation of apoptosis (via amplified ROS), ferroptosis (via GPX4 inactivation), and cuproptosis (via Cu^+^ binding to lipoylated proteins such as DLAT) [52].

The convergence of ferroptosis and cuproptosis is fundamentally orchestrated through efflux disruption, a process wherein ferroptotic signaling pathways are exploited to cripple the cellular copper export machinery. Intense LPO and oxidative stress, driven by Fe/Cu-mediated Fenton reactions, inflict catastrophic mitochondrial damage that depletes intracellular ATP stores. Since the copper-transporting P-type ATPases (such as ATP7A) are strictly ATP-dependent, this metabolic crisis directly inhibits their expression and export capacity, leading to a toxic intracellular copper surge. This accumulation acts as a catalyst for cuproptosis by inducing the proteotoxic aggregation of lipoylated DLAT [33,34,44,60]. In CRC, the copper chelator Elesclomol exemplifies this synergy; it initiates ROS-driven ferroptosis while simultaneously promoting the ubiquitin-mediated degradation of ATP7A. This loss of ATP7A ensures sustained copper retention, which subsequently triggers the degradation of System $x_{c}^{-}$ to further amplify ferroptotic cell death [60]. This mechanism is reinforced by FDX1 stabilization, where ferroptosis inducers inhibit the mitochondrial matrix proteases that typically regulate FDX1 turnover. By preventing the degradation of FDX1, these inducers stabilize the lipoylation machinery, effectively lowering the threshold for cuproptosis and ensuring a multi-pronged therapeutic attack on the cell [58].

Finally, gene editing for enhanced synergism is utilized to permanently bypass the limitations of transient metabolic inhibition. Advanced nanoplatforms now integrate CRISPR-Cas9 machinery to ensure sustained intracellular copper accumulation. For instance, RNP@Cu_2_O@SPF nanocarriers deliver CRISPR-Cas9 RNPs specifically to suppress the expression of ATP7A. This genomic knockdown of the primary copper exporter maintains high levels of cytoplasmic Cu^+^, thereby boosting CDT and driving a lethal trifecta: accelerated ferroptosis (via GSH depletion and GPX4 deactivation), intensified cuproptosis (via DLAT aggregation), and sustained ROS production [46].

## 5. Translational Perspectives and Clinical Landscape

The translation of metal-modulated RCD from preclinical models to clinical practice requires navigating a complex landscape of pharmacological and physiological hurdles. Currently, the clinical status of these interventions is bifurcated between repurposed classical chelators and novel, targeted complexes.

Initial pilot data suggested that low-dose iron chelation with DFP was well-tolerated and could reduce iron content in the dentate and caudate nuclei, with early trends hinting at motor symptom improvement [76]. However, these findings were recently challenged by a large-scale Phase 2 multicenter trial [77]. In this more robust study, DFP effectively reduced nigrostriatal iron but significantly worsened motor scores and accelerated the need for dopaminergic therapy in treatment-naive patients [77], likely due to the inhibition of iron-dependent dopamine synthesis via the depletion of iron cofactors from essential enzymes like tyrosine hydroxylase.

Regarding copper-based therapeutics, the complex Cu^II^(atsm) represents a significant translational milestone. While its neuroprotective mechanism was previously debated, recent evidence demonstrates that Cu^II^(atsm) acts as a potent inhibitor of ferroptosis, with an antiferroptotic potency approaching that of liproxstatin-1. By functioning as a lipid RTA, Cu^II^(atsm) bypasses the limitations of traditional metal sequestration, protecting neuronal cells from lipid peroxidation and ferroptotic lethality [30]. Providing a distinct translational contrast, the use of zinc-mediated copper reduction serves as a key case study for biomarker-driven neuroprotection. Recent Phase 2 clinical data [88], utilizing a CLSI-validated biomarker (Exchangeable Copper, ExcCu) for copper metabolic dysfunction [89], demonstrated that zinc therapy can achieve cognitive stabilization in pharmacodynamically responsive Alzheimer’s disease patients by modulating the free copper pool, as monitored through ceruloplasmin concentration. While these clinical assessments focus on systemic metal homeostatic restoration, the reduction of this labile copper fraction inherently mitigates the pro-oxidative catalysts required for ferroptotic initiation. Furthermore, the susceptibility to such interventions may be governed by the patient’s genetic landscape; for instance, risk alleles and haplotypes of the copper transporter ATP7B are known to modulate copper biochemistry in neurodegenerative processes [90], while the status of antioxidant enzymes like SOD1 provides a critical measure of cellular redox buffering capacity [91].

Conversely, in oncology, the history of the copper ionophore Elesclomol underscores the necessity of metabolic stratification. While the Phase 3 trial failed to meet its primary progression-free survival endpoint in the intent-to-treat population, prospectively defined subgroup analysis revealed significant clinical benefit specifically in patients with normal baseline lactate dehydrogenase (LDH) [92]. This clinical divergence is now understood to be rooted in the cellular metabolic state; recent mechanistic evidence demonstrates that Elesclomol induces copper-dependent ferroptosis via the degradation of the copper transporter ATP7A and the antioxidant stabilizer SLC7A11 [60]. Because this pro-oxidant activity is contingent upon mitochondrial respiration, the efficacy of the drug is naturally sequestered to non-hypoxic, low-LDH cell populations, suggesting that the future of metal-targeted oncology lies in biomarker-driven patient selection rather than broad application.

Despite these advancements, systemic barriers impede clinical adoption. The most prominent challenge is metal specificity to avoid off-target toxicity. For instance, in the FAIRPARK-II trial, DFP successfully reduced brain iron but caused significant clinical worsening in Parkinson’s patients, likely by depleting iron cofactors from the essential enzyme tyrosine hydroxylase [77]. Furthermore, the delivery of these agents is restricted by biological barriers: the BBB frequently excludes large or hydrophilic ferroptosis inhibitors from the central nervous system, while the dense, high-pressure TME limits the deep penetration of advanced nanomedicines [93]. Additionally, the inherent plasticity of cancer cells allows for the rapid upregulation of compensatory antioxidant networks, such as the NRF2 pathway, which can neutralize metal-induced oxidative stress [94]. Overcoming these barriers will require the development of ‘smart’ delivery systems that remain inert in systemic circulation and only release their metal-active cargo upon reaching the specific pathological environment, thereby widening the narrow therapeutic window that currently restricts the clinical utility of metal-driven cell death induction [95].

Beyond these physiological constraints, the translation of metal-targeted therapies is hindered by the absence of standardized regulatory frameworks for complex bimetallic nanopharmaceuticals. While multi-component platforms and gene-editing ionophores exhibit remarkable preclinical efficacy, their manufacturing scale-up under Good Manufacturing Practice standards presents significant reproducibility challenges regarding precise metal-to-ligand ratios and surface functionalization [96]. Moreover, transitioning from acute injury models to chronic human pathology necessitates a fundamental shift in trial endpoints; the focus must move toward establishing long-term safety profiles for continuous metal modulation and identifying reliable circulatory biomarkers, such as serum FDX1 or specific lipidomic profiles, to monitor RCD activation in real-time [97]. Only by bridging these industrial and diagnostic gaps can the promise of metal-driven cell death move from experimental validation to established clinical care.

## 6. Conclusions

The systematic exploration of metal ion homeostasis and RCD pathways has fundamentally reshaped therapeutic strategies across oncology and neurodegeneration, confirming that targeted disruption of iron and copper metabolism offers a powerful avenue for intervention. Specifically, ferroptosis, characterized by iron-dependent LPO, is established as a critical mode of cell death contributing significantly to chronic neurological disorders, such as PD and demyelination in MS, as well as acute pathologies, including hepatic and renal I/R injury. The success of tailored iron chelators, such as DFO, DFP, and the novel brain-penetrant molecule SK4, validates the strategy of mitigating oxidative stress by reducing intracellular labile iron and reversing ferroptotic markers, such as the depletion of GPX4. Finally, the identification that ferroptosis susceptibility can be modulated by non-iron metals, notably zinc through the ER transporter ZIP7, further broadens the scope of metabolic targets for neuroprotection.

In oncology, advanced therapeutic paradigms leverage the intricate crosstalk between iron-dependent ferroptosis and copper-dependent cuproptosis to bypass the adaptive resistance mechanisms of malignant cells. These synergistic strategies are increasingly realized through multifunctional bimetallic nanoconstructs, such as Fe-Cu MOFs or CuFeGA MPNs. By integrating both redox-active metals into a single delivery vehicle, these platforms facilitate the synchronized escalation of oxidative stress and the targeted disruption of metal-sensitive metabolic pathways. The amplified efficacy observed in malignancies such as CRC and TNBC stems from dual mechanisms that disrupt redox homeostasis: triggering powerful ROS generation via Fenton and Fenton-like catalysis by released Fe^2+^ and Cu^+^ ions, while simultaneously neutralizing the central anti-cell death co-inhibitor, GSH. Mechanistically, this synergistic action forms a mutually reinforcing cycle: ferroptosis induction leads to ATP depletion, subsequently inhibiting copper efflux pumps (ATP7A/7B) and causing copper overload that intensifies cuproptosis. Conversely, ferroptosis inducers like sorafenib promote cuproptosis by preventing the degradation of the lipoylation regulator FDX1 via suppressing mitochondrial proteases. Furthermore, copper stress independently promotes ferroptosis by inducing the autophagic degradation of the master ferroptosis suppressor GPX4 via its targeted autophagy receptor. A comprehensive summary of these therapeutic agents, their specific targeting categories, and their roles in clinical integration is provided in Table 4.

The translation of these mechanistic insights into effective therapies is highly promising, as evidenced by in vivo efficacy against solid tumors and localized acute injuries. NP systems exhibit precise delivery capabilities, such as T-LMD enhancing ferroptosis in TNBC xenografts, or the use of PDA NPs mitigating IVDD by concurrently scavenging ROS, chelating Fe^2+^, and suppressing GPX4 ubiquitination. Strategies combining these metal-targeting NPs with ICB effectively enhance antitumor immunity, leading to robust tumor regression and suppression of metastasis, demonstrating the clinical potential of manipulating metal-dependent RCD pathways within the broader context of cancer immunotherapy. However, as highlighted by the divergent outcomes between these preclinical successes and recent human trials, the transition to clinical care remains contingent on overcoming the narrow therapeutic window and off-target toxicities that currently restrict systemic application. Overall, the current findings confirm that targeting the molecular machinery governing metal metabolism and its crosstalk with RCD offers a critical, multi-faceted strategy for enhancing therapeutic outcomes in resistant cancers and addressing the underlying pathology of acute and chronic tissue degeneration.

## Figures and Tables

**Table 1:** Comparison of ferroptosis and cuproptosis.

Feature	Ferroptosis	Cuproptosis
Driving metal	Fe	Cu
Primary event	Lipid peroxidation (LPO)	Lipoylated protein aggregation/proteotoxic stress
Key regulator (positive)	Labile Fe^2+^	Reduced Cu^+^ (facilitated by FDX1)
Key regulator (negative)	GPX4 (using reduced GSH)	GSH (as a direct Cu^+^ chelator)
Cellular location	Cellular and organelle membranes	Mitochondrial matrix (Krebs cycle enzymes)
Diagnostic marker	GPX4 downregulation/ROS levels	FDX1-dependent DLAT oligomerization
Metabolic impact	Variable impact on ATP	Direct and severe ATP depletion

**Table 2:** Comparative analysis of metal dyshomeostasis targeting: opposing therapeutic strategies in oncology and neurodegeneration.

Feature	Cancer (Oncology)	Neurodegeneration (PD, MS)
Overall therapeutic goal	Deliberate RCD induction (therapeutic weapon)	Inhibition of RCD (therapeutic target)
Primary RCD mechanisms	Enhance ferroptosis and cuproptosis (metal toxicity)	Inhibit ferroptosis
Metabolic vulnerability	“Iron addiction” and high metabolic activity leading to susceptibility to Fe- and Cu-mediated oxidative stress.	Toxic Fe and Cu accumulation in neural tissues (e.g., Fe in PD/dopaminergic neurons, Fe/Cu in MS/oligodendrocytes).
Advanced agents	Multifunctional bimetallic nanoconstructs such as Fe/Cu metal–organic frameworks (MOFs); targeted nano-ionophores.	Synthetic iron chelators (e.g., SK4).
Homeostatic strategy	Promote fatal intracellular metal accumulation by inhibiting efflux transporters (ATP7A for Cu, FPN1 for Fe).	Sequester toxic biometals via agents designed to penetrate the blood–brain barrier (BBB) using specific transporters like large neutral amino acid transporter 1 (LAT1).
Novelty highlights	Exploiting the synergistic crosstalk between ferroptosis and cuproptosis (e.g., Cu^2+^ to Cu^+^ reduction depletes GSH to inactivate GPX4).	Targeted reversal of pathology and targeted neuroprotection through precise delivery of chelators across the BBB.

**Table 3:** Central regulatory proteins, transporters, and ion channels involved in ferroptosis and metal/redox homeostasis.

Molecule	Type	Essential Role in Ferroptosis/Homeostasis
FPN1	Iron transporter	Iron efflux: the sole known exporter of intracellular Fe^2+^. Downregulation leads to iron overload.
xCT	Amino acid transporter	Cystine uptake: the active subunit of System $x_{c}^{-}$; mediates uptake of L-cystine, the precursor for GSH.
LAT1	Amino acid transporter	Drug delivery: transports large neutral amino acids across the BBB. Exploited by agents like SK4 for CNS penetration.
SK4	K^+^ channel	Ion/volume regulation: Ca^2+^-activated K^+^ channel (KCa3.1) linked to membrane potential changes.

**Table 4:** Therapeutic agents targeting metal homeostasis and RCD.

Targeting Category	Primary Agent/System	Mechanism of Action	Target Metal(s)/Process	Therapeutic Context	Developmental Stage
I. Ferroptosis inhibition (protection)	DFO, DFP, SK4	Sequesters free labile iron via chelation to suppress Fenton-driven ROS	Fe^2+/^Fe^3+^	Neurodegeneration (PD, MS), I/R injury	DFO/DFP: Approved/Phase 2; SK4: Preclinical
	Cu^II^(atsm)	Acts as a radical trapping antioxidant (RTA) to quench lipid peroxyl radicals	Lipid peroxidation (LPO)	Neurodegeneration (ALS, PD)	Clinical trials (Phase 2)
	Zinc transporter inhibitors	Modulates ER release of Zn^2+^ to alter ferroptotic susceptibility	Zinc homeostasis	Neuroprotection	Preclinical (Proof-of-concept)
II. Synergistic induction (oncology)	Fe-Cu MOFs/MPNs	Dual ROS generation and rapid GSH depletion	Fe/Cu crosstalk	Oncology (chemoresistant tumors)	Preclinical (In vivo)
	Elesclomol	Disrupts copper efflux pumps (ATP7A) via ATP depletion	Copper efflux	Oncology (amplifying cuproptosis)	Clinically evaluated (Phase 3)
	Cu stress	Induces autophagic degradation of the master suppressor GPX4	GPX4/Ferritinophagy	Novel oncological crosstalk	Experimental (In vitro)
	Ferroptosis inducers	Stabilizes FDX1 by inhibiting mitochondrial matrix proteases	FDX1 lipoylation	Oncology (enhancing cuproptosis)	Clinically approved (Oncology)
III. Clinical integration & delivery	T-LMD magneto-liposome	Targeted iron delivery for focused ROS generation	Iron/ROS	Oncology (TNBC xenografts)	Preclinical (Xenografts)
	PDA NPs	ROS scavenging, Fe^2+^ chelation, GPX4 protection	Fe^2+^/Redox balance	Acute degeneration (IVDD)	Preclinical (Acute injury)
	NP + ICB	RCD-induced immunogenic signaling to reverse suppression	ICB	Oncology enhancement	Preclinical (Combination)

## Data Availability

No new data were created or analyzed in this study.

## Abbreviations

The following abbreviations are used in this manuscript:

ACSL4	Acyl-CoA synthetase long-chain family member 4
AD	Alzheimer’s disease
ALOX	Arachidonate lipoxygenase
ALS	Amyotrophic lateral sclerosis
ATP	Adenosine triphosphate
ATP7A	Copper-transporting ATPase 1
BBB	Blood–brain barrier
CDT	Chemodynamic therapy
CNS	Central nervous system
CRC	Colorectal cancer
Cu	Copper
CuFeGA	Copper/iron bimetal gallic acid
CuII(atsm)	Diacetylbis(N4-methylthiosemicarbazonato)copper(II)
DFO	Deferoxamine
DFP	Deferiprone
DHODH	Dihydroorotate dehydrogenase
DLAT	Dihydrolipoamide S-acetyltransferase
FDX1	Ferredoxin 1
Fe	Iron
Fer-1	Ferrostatin-1
FSP1	Ferroptosis suppressor protein 1
GA	Gallic acid
GPX4	Phospholipid hydroperoxide glutathione peroxidase 4
GSH	Glutathione
I/R	Ischemia–reperfusion
ICB	Immune checkpoint blockade
IVDD	Intervertebral disc degeneration
LAT1	Large neutral amino acid transporter 1
LDH	Lactate dehydrogenase
LH	Arachidonic acid
LIP	Labile iron pool
Lip-1	Liproxstatin-1
LOH	Lipid alcohols
LPCAT3	Lysophosphatidylcholine acyltransferase 3
LPO	Lipid peroxidation
LOOH	Lipid peroxides
METH	Methamphetamine
MOFs	Metal–organic frameworks
MPNs	Metal-phenolic networks
MPP+	1-methyl-4-phenylpyridinium
MS	Multiple sclerosis
NCOA4	Nuclear receptor coactivator 4
NP	Nanoparticle
PB	Prussian blue
PD	Parkinson’s disease
PDA	Polydopamine
PE	Phosphatidylethanolamines
PUFAs	Polyunsaturated fatty acids
RCD	Regulated cell death
RNP	Ribonucleoprotein
ROS	Reactive oxygen species
RTAs	Radical trapping antioxidants
SPF	Silica-polydopamine-folic acid
TME	Tumor microenvironment
TNBC	Triple negative breast carcinoma
xCT	The active subunit of System $x_{c}^{-}$

## References

[1] Chen Q., Li K., Li J., Liu X., Li J., Xu L., Han Y., Zou T., Wang X., Yao Y. (2025). Targeting metal ion homeostasis for regulated cell death-amplified tumor nanomedicine. Int. J. Nanomed..

[2] Gu J., Guo C., Ruan J., Li K., Zhou Y., Gong X., Shi H. (2024). From ferroptosis to cuproptosis, and calcicoptosis, to find more novel metals-mediated distinct form of regulated cell death. Apoptosis.

[3] Li Y., Du Y., Zhou Y., Chen Q., Luo Z., Ren Y., Chen X., Chen G. (2023). Iron and copper: Critical executioners of ferroptosis, cu-proptosis and other forms of cell death. Cell Commun. Signal..

[4] Hu H.T., Zhang Z.Y., Luo Z.X., Ti H.B., Wu J.J., Nie H., Yuan Z.D., Wu X., Zhang K.Y., Shi S.W. (2025). Emerging regulated cell death mechanisms in bone remodeling: Decoding ferroptosis, cuproptosis, disulfidptosis, and PANoptosis as therapeutic targets for skeletal disorders. Cell Death Discov..

[5] Wang J., Li J., Liu J., Chan K.Y., Lee H.S., Lin K.N., Wang C.C., Lau T.S. (2024). Interplay of ferroptosis and cuproptosis in cancer: Dissecting metal-driven mechanisms for therapeutic potentials. Cancers.

[6] Dixon S.J., Lemberg K.M., Lamprecht M.R., Skouta R., Zaitsev E.M., Gleason C.E., Patel D.N., Bauer A.J., Cantley A.M., Yang W.S. (2012). Ferroptosis: An iron-dependent form of nonapoptotic cell death. Cell.

[7] Imai H., Matsuoka M., Kumagai T., Sakamoto T., Koumura T. (2017). Lipid peroxidation-dependent cell death regulated by GPx4 and ferroptosis. Apoptotic Non-Apoptotic Cell Death.

[8] Mortensen M.S., Ruiz J., Watts J.L. (2023). Polyunsaturated fatty acids drive lipid peroxidation during ferroptosis. Cells.

[9] Lee N., Carlisle A.E., Peppers A., Park S.J., Doshi M.B., Spears M.E., Kim D. (2021). xCT-driven expression of GPX4 deter-mines sensitivity of breast cancer cells to ferroptosis inducers. Antioxidants.

[10] Reed A., Ichu T.A., Milosevich N., Melillo B., Schafroth M.A., Otsuka Y., Scampavia L., Spicer T.P., Cravatt B.F. (2022). LPCAT3 inhibitors remodel the polyunsaturated phospholipid content of human cells and protect from ferroptosis. ACS Chem. Biol..

[11] Kim J.W., Lee J.Y., Oh M., Lee E.W. (2023). An integrated view of lipid metabolism in ferroptosis revisited via lipidomic analysis. Exp. Mol. Med..

[12] Zhou L., Han S., Guo J., Qiu T., Zhou J., Shen L. (2022). Ferroptosis—A new dawn in the treatment of organ ischemia—Reperfusion injury. Cells.

[13] Tong X., Tang R., Xiao M., Xu J., Wang W., Zhang B., Liu J., Yu X., Shi S. (2022). Targeting cell death pathways for cancer therapy: Recent developments in necroptosis, pyroptosis, ferroptosis, and cuproptosis research. J. Hematol. Oncol..

[14] Wang F., Min J. (2021). DHODH tangoing with GPX4 on the ferroptotic stage. Signal Transduct. Target. Ther..

[15] Tang D., Kroemer G., Kang R. (2024). Targeting cuproplasia and cuproptosis in cancer. Nat. Rev. Clin. Oncol..

[16] Tang D., Chen X., Kroemer G. (2022). Cuproptosis: A copper-triggered modality of mitochondrial cell death. Cell Res..

[17] Pan C., Ji Z., Wang Q., Zhang Z., Wang Z., Li C., Lu S., Ge P. (2024). Cuproptosis: Mechanisms, biological significance, and advances in disease treatment—A systematic review. CNS Neurosci. Ther..

[18] Kaler S.G. (2011). ATP7A-related copper transport diseases—Emerging concepts and future trends. Nat. Rev. Neurol..

[19] Lukanović D., Herzog M., Kobal B., Černe K. (2020). The contribution of copper efflux transporters ATP7A and ATP7B to chemoresistance and personalized medicine in ovarian cancer. Biomed. Pharmacother..

[20] Xue Q., Yan D., Chen X., Li X., Kang R., Klionsky D.J., Kroemer G., Chen X., Tang D., Liu J. (2023). Copper-dependent au-tophagic degradation of GPX4 drives ferroptosis. Autophagy.

[21] Jakaria M., Belaidi A.A., Bush A.I., Ayton S. (2021). Ferroptosis as a mechanism of neurodegeneration in Alzheimer’s disease. J. Neurochem..

[22] Mahoney-Sánchez L., Bouchaoui H., Ayton S., Devos D., Duce J.A., Devedjian J.C. (2021). Ferroptosis and its potential role in the physiopathology of Parkinson’s Disease. Prog. Neurobiol..

[23] Xu Y., Li K., Zhao Y., Zhou L., Liu Y., Zhao J. (2023). Role of ferroptosis in stroke. Cell. Mol. Neurobiol..

[24] Wang T., Tomas D., Perera N.D., Cuic B., Luikinga S., Viden A., Barton S.K., McLean C.A., Samson A.L., Southon A. (2022). Ferroptosis mediates selective motor neuron death in amyotrophic lateral sclerosis. Cell Death Differ..

[25] Van San E., Debruyne A.C., Veeckmans G., Tyurina Y.Y., Tyurin V.A., Zheng H., Choi S.M., Augustyns K., van Loo G., Michalke B. (2023). Ferroptosis contributes to multiple sclerosis and its pharmacological targeting sup-presses experimental disease progression. Cell Death Differ..

[26] Hu S., Huang X., Huang J., Qian Y., Tian Y., Xiao Y., Qi X., Zhou X., Yang Z., Chen Z. (2023). Iron chelation prevents nigro-striatal neurodegeneration in a chronic methamphetamine mice model. Neurotoxicology.

[27] Jhelum P., Santos-Nogueira E., Teo W., Haumont A., Lenoël I., Stys P.K., David S. (2020). Ferroptosis mediates cu-prizone-induced loss of oligodendrocytes and demyelination. J. Neurosci..

[28] Jia H., Liu X., Cao Y., Niu H., Zhang L., Li R., Li F., Sun D., Shi M., Wa L. (2023). Deferoxamine ameliorates neuro-logical dysfunction by inhibiting ferroptosis and neuroinflammation after traumatic brain injury. Brain Res..

[29] Fiorillo M., Tóth F., Brindisi M., Sotgia F., Lisanti M.P. (2020). Deferiprone (DFP) targets cancer stem cell (CSC) propagation by inhibiting mitochondrial metabolism and inducing ROS production. Cells.

[30] Southon A., Szostak K., Acevedo K.M., Dent K.A., Volitakis I., Belaidi A.A., Barnham K.J., Crouch P.J., Ayton S., Don-nelly P.S. (2020). CuII (atsm) inhibits ferroptosis: Implications for treatment of neurodegenerative disease. Br. J. Pharmacol..

[31] Chen K., Zhou A., Zhou X., He J., Xu Y., Ning X. (2024). Cellular Trojan Horse initiates bimetallic Fe-Cu MOF-mediated syner-gistic cuproptosis and ferroptosis against malignancies. Sci. Adv..

[32] Shen Q.H., Yu L.B., Yao S.M., Li Z.Y., Zhang Q.Q., Wang P., Tan C.P. (2025). Bioinspired construction of Cu–Fe bimetallic mul-tienzyme mimetics as co-inducers of cuproptosis and ferroptosis. J. Med. Chem..

[33] Xu W., Qian J., Hou G., Wang T., Wang J., Wang Y., Yang L., Cui X., Suo A. (2022). A hollow amorphous bimetal organic framework for synergistic cuproptosis/ferroptosis/apoptosis anticancer therapy via disrupting intracellular redox ho-meostasis and copper/iron metabolisms. Adv. Funct. Mater..

[34] Zhang X., Zhu J., Wang S., Li S., E J., Hu J., Mou R., Ding H., Yang P., Xie R. (2024). A copper/ferrous-engineering redox ho-meostasis disruptor for cuproptosis/ferroptosis Co-activated nanocatalytic therapy in liver cancer. Adv. Funct. Mater..

[35] Lachowicz J.I., Pichiri G., Piludu M., Fais S., Orrù G., Congiu T., Piras M., Faa G., Fanni D., Dalla Torre G. (2022). Thymosin β4 is an endogenous iron chelator and molecular switcher of ferroptosis. Int. J. Mol. Sci..

[36] Jhelum P., David S. (2022). Ferroptosis: Copper-iron connection in cuprizone-induced demyelination. Neural Regen. Res..

[37] Li Y., Liu J., Chen Y., Weichselbaum R.R., Lin W. (2024). Nanoparticles synergize ferroptosis and cuproptosis to potentiate cancer immunotherapy. Adv. Sci..

[38] Gutbier S., Kyriakou S., Schildknecht S., Ückert A.K., Brüll M., Lewis F., Dickens D., Pearson L., Elson J.L., Michel S. (2020). Design and evaluation of bi-functional iron chelators for protection of dopaminergic neurons from toxicants. Arch. Toxicol..

[39] Zeng X., An H., Yu F., Wang K., Zheng L., Zhou W., Bao Y., Yang J., Shen N., Huang D. (2021). Benefits of iron chelators in the treatment of Parkinson’s disease. Neurochem. Res..

[40] Qin D., Li D., Wang C., Guo S. (2023). Ferroptosis and central nervous system demyelinating diseases. J. Neurochem..

[41] Zhu Y., Zhang J., Deng Q., Chen X. (2024). Mitophagy-associated programmed neuronal death and neuroinflammation. Front. Immunol..

[42] Shen Y., Li X., Dong D., Zhang B., Xue Y., Shang P. (2018). Transferrin receptor 1 in cancer: A new sight for cancer therapy. Am. J. Cancer Res..

[43] Xie Y., Jiang T., Wang J., Qian Y., Xu W., Zhao G., Gao H., Li C. (2025). EDTA-Mg nano-chelators amplify ferroptosis by arti-ficially simulating the epithelial-mesenchymal transition process and endogenous iron deprivation. Angew. Chem..

[44] Yin W., Chang J., Zhi H., Chen S., Xue L., Zhang X., Dong H., Li Y. (2025). Copper deposited metal-phenolic networks enabled crosstalk between cuproptosis and ferroptosis for triple-negative breast cancer immunotherapy. Chem. Eng. J..

[45] Yan X., Liu H., Guo L., Liu C., Zhang S., Wang X., Tang Y., Zhou R., Jiang X., Wang E. (2025). Multifunctional drug delivery nanoparticles for combined chemotherapy/chemodynamic/photothermal therapy against colorectal cancer through synergistic cuproptosis/ferroptosis/apoptosis. Mater. Today Bio.

[46] Wu X., Bai Z., Wang H., Wang H., Hou D., Xu Y., Wo G., Cheng H., Sun D., Tao W. (2024). CRISPR-Cas9 gene editing strengthens cuproptosis/chemodynamic/ferroptosis synergistic cancer therapy. Acta Pharm. Sin. B.

[47] Gynther M., Puris E., Peltokangas S., Auriola S., Kanninen K.M., Koistinaho J., Huttunen K.M., Ruponen M., Vellonen K.S. (2018). Alzheimer’s disease phenotype or inflammatory insult does not alter function of L-type amino acid transporter 1 in mouse blood-brain barrier and primary astrocytes. Pharm. Res..

[48] Abdelaal G., Carter A., Panayiotides M.I., Tetard D., Veuger S. (2022). Novel iron chelator SK4 demonstrates cytotoxicity in a range of tumour derived cell lines. Front. Mol. Biosci..

[49] Tian H., Xiong Y., Zhang Y., Leng Y., Tao J., Li L., Qiu Z., Xia Z. (2022). Activation of NRF2/FPN1 pathway attenuates myo-cardial ischemia–reperfusion injury in diabetic rats by regulating iron homeostasis and ferroptosis. Cell Stress Chaperones.

[50] Yanagiya R., Miyatake Y., Watanabe N., Shimizu T., Kanamori A., Ueno M., Okabe S., Carreras J., Nakayama S., Hasegawa A. (2024). Amino acid influx via LAT1 regulates iron demand and sensitivity to PPMX-T003 of aggres-sive natural killer cell leukemia. Leukemia.

[51] Manfroni G., Ragonese F., Monarca L., Astolfi A., Mancinelli L., Iannitti R.G., Bastioli F., Barreca M.L., Cecchetti V., Fioretti B. (2020). New insights on KCa3.1 channel modulation. Curr. Pharm. Des..

[52] Zhao F., Yu H., Wang C., Xu J., Gao H., Ying Y., Li W., Li J., Zheng J., Qiao L. (2024). Copper/iron bimetal phenolic networks boosted apoptosis/ferroptosis/cuproptosis combined tumor therapy through dual glutathione depletion. Chem. Eng. J..

[53] Liu M.R., Zhu W.T., Pei D.S. (2021). System Xc−: A key regulatory target of ferroptosis in cancer. Investig. New Drugs.

[54] Tu K., Deng H., Kong L., Wang Y., Yang T., Hu Q., Hu M., Yang C., Zhang Z. (2020). Reshaping tumor immune microenvi-ronment through acidity-responsive nanoparticles featured with CRISPR/Cas9-mediated programmed death-ligand 1 attenuation and chemotherapeutics-induced immunogenic cell death. ACS Appl. Mater. Interfaces.

[55] Gu L., Sun Y., Bai T., Shao S., Tang S., Xue P., Cai W., Qin X., Zeng X., Yan S. (2025). Functional nanozyme system for syner-gistic tumor immunotherapy via cuproptosis and ferroptosis activation. J. Nanobiotechnol..

[56] Matsui M.S., Petris M.J., Niki Y., Karaman-Jurukovska N., Muizzuddin N., Ichihashi M., Yarosh D.B. (2015). Omeprazole, a gastric proton pump inhibitor, inhibits melanogenesis by blocking ATP7A trafficking. J. Investig. Dermatol..

[57] Liang X., Fang S., Xin Y., Lei J., Wang W., Wei Y., Li W., Li C., Tang H., Wei X. (2025). Cascade-targeting copper homeostasis nano-regulators for mild-photothermal boosted cuproptosis/ferroptosis mediated breast cancer therapy. J. Nanobiotechnol..

[58] Wang W., Lu K., Jiang X., Wei Q., Zhu L., Wang X., Jin H., Feng L. (2023). Ferroptosis inducers enhanced cuproptosis induced by copper ionophores in primary liver cancer. J. Exp. Clin. Cancer Res..

[59] Yu Z., Song L., Wang Y., Chen X., Chen P., Zhong S., Li Y., Tang L. (2024). Integrating bulk-RNA and single-cell analysis re-veals heterogeneous expression of cuproptosis-related sorafenib-resistant genes in hepatocellular carcinoma. Oncologie.

[60] Gao W., Huang Z., Duan J., Nice E.C., Lin J., Huang C. (2021). Elesclomol induces copper-dependent ferroptosis in colorectal cancer cells via degradation of ATP7A. Mol. Oncol..

[61] Chen P.H., Wu J., Xu Y., Ding C.K.C., Mestre A.A., Lin C.C., Yang W.H., Chi J.T. (2021). Zinc transporter ZIP7 is a novel deter-minant of ferroptosis. Cell Death Dis..

[62] Zhang C., Liu Z., Zhang Y., Ma L., Song E., Song Y. (2020). “Iron free” zinc oxide nanoparticles with ion-leaking properties disrupt intracellular ROS and iron homeostasis to induce ferroptosis. Cell Death Dis..

[63] Gao G., Xia H., Shi J., Zheng P., Wu W., Wu S., Qi T., Song H., Gu Y., Li J. (2025). Carbon dot nanozymes with ferrous ion-chelating and antioxidative activity inhibiting ferroptosis to alleviate renal ischemia-reperfusion injury. Small.

[64] Yang X., Chen Y., Guo J., Li J., Zhang P., Yang H., Rong K., Zhou T., Fu J., Zhao J. (2023). Polydopamine nanoparticles target-ing ferroptosis mitigate intervertebral disc degeneration via reactive oxygen species depletion, iron ions chelation, and GPX4 ubiquitination suppression. Adv. Sci..

[65] Shetake N.G., Das S.K., Kumar A., Pandey B.N. (2024). Nano-inducer of ferroptosis for targeted chemotherapy of human triple negative breast carcinoma. Biomater. Adv..

[66] Zou J.X., Chang M.R., Kuznetsov N.A., Kee J.X., Babak M.V., Ang W.H. (2025). Metal-based immunogenic cell death inducers for cancer immunotherapy. Chem. Sci..

[67] Yamada N., Karasawa T., Wakiya T., Sadatomo A., Ito H., Kamata R., Watanabe S., Komada T., Kimura H., Sana-da Y. (2020). Iron overload as a risk factor for hepatic ischemia-reperfusion injury in liver transplantation: Potential role of ferroptosis. Am. J. Transplant..

[68] Huang X., Wang Y., Cui X., Yan Q., Xue T., Jing X. (2025). Insight into myocardial ischemia-reperfusion injury from the per-spective of ferroptosis. Perfusion.

[69] Zhao H., Ji Q.H., Jia Z.Z., Shen L.H. (2025). Association between deep gray matter iron deposition and clinical symptoms in Parkinson’s disease: A quantitative susceptibility mapping study. Front. Neurol..

[70] Devos D., Cabantchik Z.I., Moreau C., Danel V., Mahoney-Sanchez L., Bouchaoui H., Gouel F., Rolland A.S., Duce J.A., Devedjian J.C. (2020). Conservative iron chelation for neurodegenerative diseases such as Parkinson’s disease and amyotrophic lateral sclerosis. J. Neural Transm..

[71] Rouault T.A. (2013). Iron metabolism in the CNS: Implications for neurodegenerative diseases. Nat. Rev. Neurosci..

[72] Zheng Y., Tan X., Wang X., Mao R., Guo J. (2025). Targeting ferroptosis with natural products in stroke: Therapeutic mecha-nisms and translational opportunities. Front. Pharmacol..

[73] Chun H.S., Gibson G.E., DeGiorgio L.A., Zhang H., Kidd V.J., Son J.H. (2001). Dopaminergic cell death induced by MPP+, ox-idant and specific neurotoxicants shares the common molecular mechanism. J. Neurochem..

[74] Yee A.G., Lee S.M., Hunter M.R., Glass M., Freestone P.S., Lipski J. (2014). Effects of the Parkinsonian toxin MPP+ on electro-physiological properties of nigral dopaminergic neurons. Neurotoxicology.

[75] Negida A., Hassan N.M., Aboeldahab H., Zain Y.E., Negida Y., Cadri S., Cadri N., Cloud L.J., Barrett M.J., Berman B. (2024). Efficacy of the iron-chelating agent, deferiprone, in patients with Parkinson’s disease: A systematic review and meta-analysis. CNS Neurosci. Ther..

[76] Martin-Bastida A., Ward R.J., Newbould R., Piccini P., Sharp D., Kabba C., Patel M.C., Spino M., Connelly J., Tricta F. (2017). Brain iron chelation by deferiprone in a phase 2 randomised double-blinded placebo controlled clinical trial in Parkinson’s disease. Sci. Rep..

[77] Devos D., Labreuche J., Rascol O., Corvol J.C., Duhamel A., Guyon Delannoy P., Poewe W., Compta Y., Pavese N., Růžička E. (2022). Trial of deferiprone in Parkinson’s disease. N. Engl. J. Med..

[78] Abdelaal G., Carter A., Cheung W., Panayiotidis M., Racey S., Tétard D., Veuger S. (2023). Novel iron chelator SK4 drives cytotoxicity through inhibiting mitochondrial metabolism in ovarian and triple negative breast cancer cell lines. Biomedicines.

[79] Ahmed H.S. (2025). The multifaceted role of L-type amino acid transporter 1 at the blood–brain barrier: Structural implications and therapeutic potential. Mol. Neurobiol..

[80] Barić N., Cetina M. (2017). Initial bonding of ferric ion (Fe^3+^) and amyloid beta (Aß) peptide as the precondition for oxidative stress in Alzheimer’s disease. Glycative Stress Res..

[81] Bruni A., Pepper A.R., Pawlick R.L., Gala-Lopez B., Gamble A.F., Kin T., Seeberger K., Korbutt G.S., Bornstein S.R., Linkermann A. (2018). Ferroptosis-inducing agents compromise in vitro human islet viability and function. Cell Death Dis..

[82] Dusek P., Hofer T., Alexander J., Roos P.M., Aaseth J.O. (2022). Cerebral iron deposition in neurodegeneration. Biomolecules.

[83] Skjørringe T., Møller L.B., Moos T. (2012). Impairment of interrelated iron-and copper homeostatic mechanisms in brain con-tributes to the pathogenesis of neurodegenerative disorders. Front. Pharmacol..

[84] Siotto M., Simonelli I., Pasqualetti P., Mariani S., Caprara D., Bucossi S., Ventriglia M., Molinario R., Antenucci M., Rongioletti M. (2016). Association between serum ceruloplasmin specific activity and risk of Alzheimer’s disease. J. Alzheimers Dis..

[85] Taraboletti A., Walker T., Avila R., Huang H., Caporoso J., Manandhar E., Leeper T.C., Modarelli D.A., Medicetty S., Shriver L.P. (2017). Cuprizone intoxication induces cell intrinsic alterations in oligodendrocyte metabolism independent of copper chelation. Biochemistry.

[86] Liu S., Gao X., Zhou S. (2022). New target for prevention and treatment of neuroinflammation: Microglia iron accumulation and ferroptosis. ASN Neuro.

[87] Shinada M., Suzuki H., Hanyu M., Igarashi C., Matsumoto H., Takahashi M., Hihara F., Tachibana T., Sogawa C., Zhang M.R. (2023). Trace Metal Impurities Effects on the Formation of [64Cu] Cu-diacetyl-bis (N 4-methylthiosemicarbazone)([64Cu] Cu-ATSM). Pharmaceuticals.

[88] Squitti R., Benussi A., Fostinelli S., Geviti A., Rivolta J., Ventriglia M., Micera A., Rongioletti M., Ghidoni R., Santilli M. (2025). Zinc Therapy in Mild Cognitive Impairment: Cognitive Stabilization in Pharmacodynamically Responsive Patients in the ZINCAiD Trial. Biomolecules.

[89] Squitti R., Pal A., Ivanova I.D., Marianetti M., Rongioletti M. (2025). CLSI Validation of Exchangeable Copper Determination in Serum by ICP-MS: A Focus on Alzheimer’s Disease and Wilson Disease. Biomolecules.

[90] Squitti R., Siotto M., Arciello M., Rossi L. (2016). Non-ceruloplasmin bound copper and ATP7B gene variants in Alzheimer’s disease. Metallomics.

[91] Rossi L., Squitti R., Pasqualetti P., Marchese E., Cassetta E., Forastiere E., Rotilio G., Rossini P.M., Finazzi-Agró A. (2002). Red blood cell copper, zinc superoxide dismutase activity is higher in Alzheimer’s disease and is decreased by D-penicillamine. Neurosci. Lett..

[92] O’Day S.J., Eggermont A.M., Chiarion-Sileni V., Kefford R., Grob J.J., Mortier L., Robert C., Schachter J., Testori A., Mackiewicz J. (2013). Final results of phase III SYMMETRY study: Randomized, double-blind trial of elesclomol plus paclitaxel versus paclitaxel alone as treatment for chemotherapy-naive patients with advanced melanoma. J. Clin. Oncol..

[93] Wang Y., Sun T., Jiang C. (2022). Nanodrug delivery systems for ferroptosis-based cancer therapy. J. Control. Release.

[94] Anandhan A., Dodson M., Schmidlin C.J., Liu P., Zhang D.D. (2020). Breakdown of an ironclad defense system: The critical role of NRF2 in mediating ferroptosis. Cell Chem. Biol..

[95] Liu Q., Zhao Y., Zhou H., Chen C. (2023). Ferroptosis: Challenges and opportunities for nanomaterials in cancer therapy. Regen. Biomater..

[96] Zheng C., Li M., Ding J. (2021). Challenges and opportunities of nanomedicines in clinical translation. Bio Integr..

[97] Razaghi A., Heimann K., Schaeffer P.M., Gibson S.B. (2015). Negative regulators of cell death pathways in cancer: Perspective on biomarkers and targeted therapies. Apoptosis.

